# Monacyclinones, New Angucyclinone Metabolites Isolated from *Streptomyces* sp. M7_15 Associated with the Puerto Rican Sponge *Scopalina ruetzleri*

**DOI:** 10.3390/md13084682

**Published:** 2015-07-29

**Authors:** Jan Vicente, Allison K. Stewart, Ryan M. van Wagoner, Elizabeth Elliott, Andrea J. Bourdelais, Jeffrey L. C. Wright

**Affiliations:** 1 Center for Marine Science, University of North Carolina Wilmington, 5600 Marvin K. Moss Lane Wilmington, NC 28409, USA; E-Mails: jvicente@umces.edu (J.V.); stewarta@uncw.edu (A.K.S.); r.m.vanwagoner@pham.utah.edu (R.M.W.); elliotte@uncw.edu (E.E.); bourdelaisa@uncw.edu (A.J.B.); 2 Institute of Marine and Environmental Technology, Center for Environmental Science, University of Maryland, 701 E Pratt St Suite 236, Baltimore, MD 21202, USA

**Keywords:** angucyclinone, *Streptomyces*, antibiotic, anticancer

## Abstract

During an investigation of new actinomycete species from *Caribbean sponges* for novel bioactive natural products, frigocyclinone (**1**), dimethyldehydrorabelomycin (**3**) and six new angucyclinone derivatives were isolated from *Streptomyces* sp. strain M7_15 associated with the sponge *Scopalina ruetzleri*. Of these, monacyclinones A–B (**4**–**5**) contain the core ring structure of dehydrorabelomycin (**2**) with the aminodeoxysugar found in frigocyclinone (**1**). Monacyclinone C (**6**) is a hydroxylated variant of frigocyclinone (**1**) and monacyclinone D (**7**) is a Baeyer Villiger derivative of (**6**) which also exists as the open chain hydrolysis product monacyclinone E (**8**). Monacyclinone F (**9**) contains two unique epoxide rings attached to the angucyclinone moiety and an additional aminodeoxysugar attached through an angular oxygen bond. All structures were confirmed through spectral analyses. Activity against rhabdomycosarcoma cancer cells (SJCRH30) after 48 h of treatment was observed with frigocyclinone (**1**; EC_50_ = 5.2 µM), monacyclinone C (**6**; 160 µM), monacyclinone E (**8**; 270 µM), and monacyclinone F (**9**; 0.73 µM). The strongest bioactivity against rhabdomycosarcoma cancer cells and gram-positive bacteria was exhibited by compound **9**, suggesting that the extra aminodeoxysugar subunit is important for biological activity.

## 1. Introduction

Marine sponges harbor a highly complex diversity of microorganisms including a variety of the pharmaceutically valuable *Streptomyces* sp. [1,2,3,4,5]. Sponge-associated *Streptomyces* sp. are dominant producers of aromatic polyketides such as anthracyclines, tetracyclines and angucyclinones that contain a benzene-anthraquinone moiety [6,7]. Over 100 angucyclinone derivatives have been discovered thus far as a result of a variety of oxygenation, glycosylation, and dehydration reactions [8,9,10,11]. These derivatives have all shown a wide range of biological activities (*i.e*., anti-tumor, antifungal, antiviral, antimicrobial) [12,13,14].

Due to the promising diversity of sponge associated *Streptomyces* sp. and their ability to produce secondary metabolites, sponges in the Caribbean were screened for the presence of *Streptomyces* sp. A unique isolate (*Streptomyces* sp. M7_15) was cultivated from the Puerto Rican sponge *Scopalina ruetzleri* [15] collected from Mona Island [16]. The main compound produced by *Streptomyces* sp. M7_15 was found to be the angucyclinone derivative frigocyclinone which was previously isolated from a *Streptomyces griseus* strain cultured from the soils of Antarctica [17].

**Figure 1:**
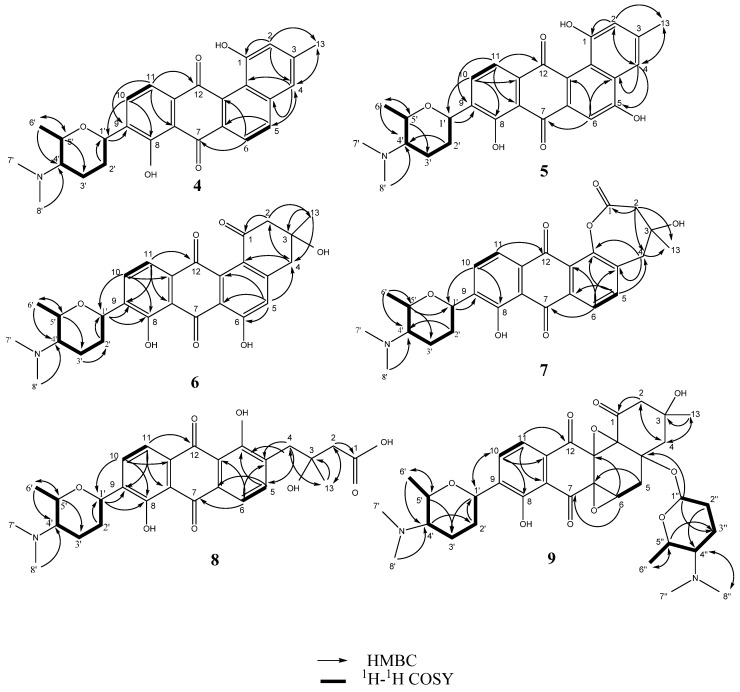
Key ^1^H–^1^H COSY and HMBC correlations of compounds **4**–**9**.

Angucyclinone natural products have been isolated from a variety of actinobacteria since the discovery of tetrangomycin and tetrangulol in the 1960s, and congeners of this increasingly diverse natural product class have exhibited intriguing activity profiles [10,11,18]. Frigocyclinone (**1**) was the first angucyclinone derivative to have a *C*-glycosidic linked aminodeoxy sugar moiety (Figure 1). In this study we report six related derivatives, called the monacyclinones, which contain similar features to frigocyclinone including the characteristic *C*-glycosidic aminodeoxy sugar moiety. Significantly, the wild-type strain also produces monacyclinone D (**7**) in which the A ring is found as a lactone moiety, which most likely arises by a Baeyer-Villiger reaction. In addition, this compound co-occurs with the open chain hydrolysis product monacyclinone E (**8**). Monacyclinone F (**9**) contains two epoxide rings attached to the angucyclinone moiety and an additional aminodeoxysugar unit, but this time attached by an ether linkage (Figure 1). These novel compounds displayed biological activity against human rhabdomyosarcoma cancer cells (SJCRH30) and gram-positive bacteria.

## 2. Results and Discussion

### 2.1. Purification and Structure Elucidation of Angucyclinone Derivatives

Frigocyclinone (**1**) was previously isolated and characterized from a species of *Streptomyces griseus* isolated from soils of Antarctica [17]. This compound has a very unusual *C*-glycosidic linked aminodeoxy sugar moiety attached to an anthraquinone chromophore that displays a characteristic UV absorption spectrum. Upon analysis of the liquid chromatography-diode array detection-electrospray ionization mass spectrometry (LC-DAD-ESIMS) data of fractions from *Streptomyces* sp. strain M7_15, the 80%–100% MeOH fractions showed a UV absorbance spectrum similar to frigocyclinone (287, 314, and 405 nm). Further purification of this fraction yielded 13 mg of a compound with a molecular weight of 463 Da. Structural characterization of this compound following ^1^H NMR and ^13^C NMR 1D and 2D experiments established that the ^1^H NMR and ^13^C NMR signals were consistent with those reported for frigocyclinone (Table S1). In addition, high-resolution electrospray ionization mass spectrometry (HRESIMS) showed a pseudomolecular ion at *m*/*z* 464.2061 [M + H]^+^ suggesting a molecular formula of C_27_H_30_NO_6_ (calculated: 464.2073, Δ_m_ = −2.6 ppm), consistent with (**1**).

Dimethyldehydrorabelomycin (**3**, 3.2 mg) was isolated as a red amorphous powder from the frigocyclinone-containing fraction. An HRESIMS measurement of [M + H]^+^ at *m*/*z* 349.1071 suggested the molecular formula C_21_H_17_O_5_ (calculated: *m*/*z* 349.1076, Δ_m_ = −1.4 ppm) indicating 14 double bond equivalents (Table S2). The UV spectrum of compound **3** in pyridine exhibited maximum absorbance at 290, 318, and 415 nm, indicative of a substituted anthraquinone moiety. Compound **3** was previously obtained by exposing dehydrorabelomycin (**2**) [19], to the methyltransferase enzyme GilMT [20]. Comparison of all ^1^H NMR and ^13^C NMR data for **3** were in agreement with those reported by Tibrewal *et al.* [20] (Table S2).

**Figure 2:**
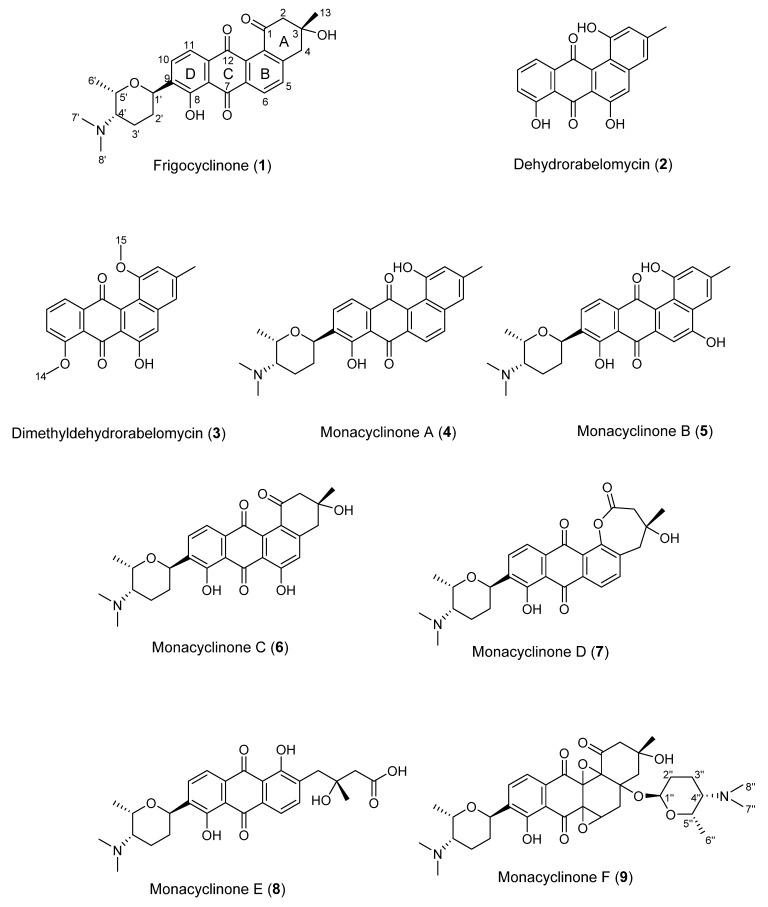
Monacyclinone derivatives isolated from *Streptomyces* sp. M7_15 including previously described frigocyclinone and dehydrorabelomycin.

**Figure 3:**
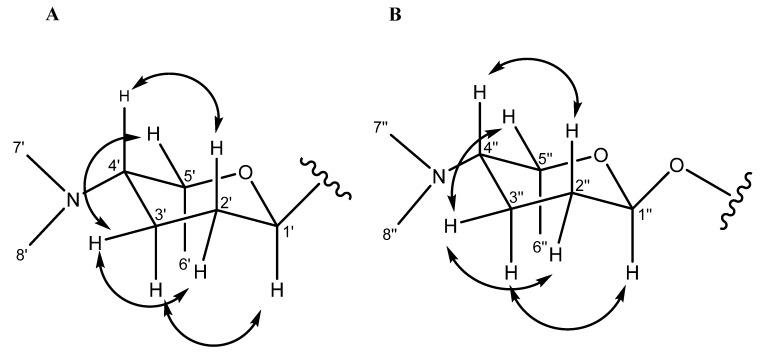
ROESY correlations for the *C*-linked aminodeoxysugar of compounds **4**–**8** (**A**) ROESY correlations for the *O*-linked aminodeoxysugar of compound **9** (**B**).

Monacyclinone A (**4**, 4.3 mg) was isolated as a yellow powder and was calculated to have a molecular formula of C_27_H_28_NO_5_ ([M + H]^+^ observed: *m*/*z* 446.1974, calculated: *m*/*z* 446.1967, Δ_m_ = 1.6 ppm) as determined by HRESIMS, and corresponding to an additional double bond equivalent compared with **1** (Figure 2), and a possible loss of H_2_O. The UV spectrum of compound **4** in pyridine exhibited maximum absorbance at 280, 315, and 422 nm. Analysis of the ^1^H and ^13^C NMR spectral data for **4** (Table 1) showed many similarities with the chemical shift values corresponding to the frigocyclinone sugar moiety, which was attached to the aromatic nucleus at C-9 based on an HMBC correlation between H-1′ and C-9 (Figure 1 and Table S3). Resonances for the two pairs of aromatic protons on the B ring (H-5 δ_H_ 8.19 d, *J* = 8 Hz; H-6 δ_H_ 8.40 d, *J* = 8 Hz) and the D ring (H-10 δ_H_ 8.03 d, *J* = 8 Hz; H-11 δ_H_ 7.91 d, *J* = 8 Hz) were consistent with those observed in the ^1^H spectrum of **1** (Table 1, Figure 2). The chemical shifts of the A ring aromatic protons (H-2 δ_H_ 7.33 s; H-4 δ_H_ 7.28 s) were consistent with those of dehydrorabelomycin (**2**). The assignment of the relative configuration at carbons C-1′, C-4′, C-5′ was based on intense ROESY cross peaks between protons H-1′ (δ_H_ 5.26_ax_), H-3′ at (δ_H_ 1.98_ax_), and H-6′ at (δ_H_ 1.52_ax_) which positions these protons on the same side of the angucyclinone moiety (Table S3; Figure 3). Additionally, ROESY cross peaks between proton H-2′_ax_ (δ_H_ 1.44) and H-4′_ax_ at (δ_H_ 2.82) indicated diaxial interactions on the opposite face of the sugar ring (Figure 3).

Monacyclinone B (**5**, 7.4 mg) was isolated as an orange powder and was calculated to have a molecular formula of C_27_H_28_NO_6_ ([M + H]^+^ observed: *m*/*z* 462.1917, calculated: *m*/*z* 462.1917, Δ_m_ 0.0 ppm) corresponding to an additional oxygen compared with **4**, and shared a similar UV absorption spectrum (299, 328, and 445 nm). The difference in molecular formulae suggested **5** contained an additional phenolic group as indicated by the appearance of a new quaternary carbon shift at δ 157.4 in the ^13^C NMR spectrum (Table 1). An HMBC correlation from the singlet aromatic proton at (H-6, δ_H_ 8.01) to C-4a (δ_C_ 124.2) and C-7 (δ_C_ 189.9) implied that the phenolic substituent resided on C-5, and further evidenced by the upfield shift of the aromatic proton at C-6 (δ_C_ 105.6) in **5** when compared to **4**. Comparison of the ^1^H and ^13^C NMR signals for the rest of the molecule with those observed for **4** supported the proposed structure (Table S4), including the presence of the aminodeoxy sugar with identical configuration to the corresponding moiety in **4**.

The molecular formula of monacyclinone C (**6**, 1.5 mg) (C_27_H_30_NO_7_ [M + H]^+^, observed: *m*/*z* 480.2027, calculated: *m*/*z* 480.2022, Δ_m_ = 1.0 ppm) indicated 14 double bond equivalents and an additional oxygen atom compared with frigocyclinone (**1**) (Figure 2). This was consistent with the UV spectrum of **6** which showed absorption maxima at 280, 301 and 429 in accordance with the UV absorption profile of frigocyclinone (**1**). Much of the 1D and 2D NMR data for **6** was identical to **1**: For example, the characteristic C-1 carbonyl shift at δ_C_ 196.9, as well as HMBC correlations between the two methylene groups at C-2 (CH_2_ δ_H_ 3.22 m, 2H) and C-4 (CH_2_ δ_H_ 3.25 m, 2H) with the methyl substituent at C-3 (δ_H_ 1.56 s, 3H; δ_C_ 30.1) (Figure 3), indicated that ring A was unchanged (Table 1, Figure 2). In addition, the usual 1D and 2D NMR data established an aminodeoxysugar group attached to C-9 of the anthraquinone moiety (Table S5). Similar to monacyclinone B (**5**), the ^1^H NMR data of **6** (Table 1) showed the loss of an aryl proton, while the ^13^C NMR spectrum now contained a quaternary carbon at δ_C_ 164.5 consistent with a phenolic substituent. However in contrast to **5**, HMBC correlations in the B ring of **6** were observed from the singlet aromatic proton H-5 (δ_H_ 7.16) to C-4 (δ_C_ 45.0) and C-6 (δ_C_ 164.5), indicating that the phenolic group resided on C-6.

**Table 1:** ^13^C and ^1^H NMR data for compounds **4**, **5**, **6**, **7**, **8** (in pyridine-*d*_5_).

Position	Monacyclinone A (4)		Monacyclinone B (5)		Monacyclinone C (6)		Monacyclinone D (7)		Monacyclinone E (8)	
	^13^C	^1^H (m, *J* Hz)	^13^C	^1^H (m, *J* Hz)	^13^C	^1^H (m, *J* Hz)	^13^C	^1^H (m, *J* Hz)	^13^C	^1^H (m, *J* Hz)
1	156.7		164.8		196.9		172.9		175.7	
2	119.9	7.33 (s)	116.0	8.22 (s)	54.5	3.22 (m)	45.3	3.23 (d, 17.0)	46.9	3.02 (m)
						3.22 (m)		3.21 (d, 17.0)		3.02 (m)
3	142.6		141.5		71.9		90.7		72.4	
4	121.6	7.28 (s)	121.0	7.43 (s)	45.0	3.25 (m)	40.3	3.21 (d, 17.0)	41.0	2.52 (m)
						3.25 (m)		3.81 (d, 17.0)		3.47 (m)
4a	120.3		124.2		153.6		139.4		136.7	
5	137.5	8.19 (d, 8.0)	157.4		122.4	7.16 (s)	130.5	7.53 (d, 8.0)	140.7	8.06 (d, 8.0)
6	122.5	8.40 (d, 8.0)	105.6	8.01 (s)	164.5		120.8	8.01 (d, 8.0)	118.9	7.90 (d, 8.0)
6a	135.6		123.5		117.5		133.4		132.4	
7	188.1		189.9		193.1		189.6		188.9	
7a	115.0		115.2		115.4		115.9		116.3	
8	158.7		158.9		158.8		159.2		160.0	
9	140.0		139.9		139.0		138.2		141.0	
10	134.1	8.03 (d, 8.0)	134.0	8.05 (d, 8.0)	134.5	8.03 (d, 8.0)	133.7	8.09 (d, 8.0)	134.0	8.13 (d, 8.0)
11	121.4	7.91 (d, 8.0)	121.3	7.97 (d, 8.0)	119.5	7.82 (d, 8.0)	119.0	8.05 (d, 8.0)	119.6	8.00 (d, 8.0)
11a	134.6		135.1		135.3		133.4		132.6	
12	189.5		187.5		184.1		181.0		189.1	
12a	134.3		132.2		130.6		116.7		116.3	
12b	121.1		123.7		122.6		160.0		162.6	
13	21.5	2.39 (s)	21.7	2.46 (s)	30.1	1.56 (s)	27.3	1.78 (s)	27.8	1.69 (s)
1′	65.2	5.26_ax_ (d, 11.0)	65.2	5.23_ax_ (m)	65.1	5.22_ax_ (dd, 11.0,1.0)	64.7	5.26_ax_ (m)	65.2	5.25_ax_ (m)
2′	32.5	1.44_ax_ (m)	32.5	1.44_ax_ (m)	32.1	1.36_ax_ (m)	31.9	1.39_ax_ (m)	32.2	1.42_ax_ (m)
		2.39_eq_ (m)		2.30_eq_ (m)		2.34_eq_ (m)		2.33_eq_ (m)		2.33_eq_ (m)
3′	22.4	1.98_ax_ (m)	22.8	1.79_ax_ (m)	22.7	1.81_ax_ (m)	22.5	1.83_ax_ (m)	23.1	1.69_ax_ (m)
		1.98_eq_ (m)		1.93_eq_ (m)		1.94_eq_ (m)		1.94_eq_ (m)		1.89_eq_ (m)
4′	64.7	2.82_ax_ (br s)	64.5	2.53_ax_ (s)	64.7	2.53_ax_ (m)	64.2	2.54_ax_ (m)	64.7	2.33_ax_ (m)
5′	71.2	4.72 (m)	71.6	4.64 (m)	71.9	4.61 (m)	71.8	4.63 (m)	72.0	4.61 (m)
6′	12.4	1.52_ax_ (d, 7.0)	12.2	1.44_ax_ (m)	12.3	1.44_ax_ (d, 7.0)	12.0	1.46_ax_ (d, 7.0)	12.3	1.42_ax_ (m)
7′	42.8	2.52 (s)	43.2	2.31 (s)	43.0	2.34 (s)	43.0	2.33 (s)	43.3	2.19 (s)
8′	42.8	2.52 (s)	43.2	2.31 (s)	43.0	2.34 (s)	43.0	2.33 (s)	43.3	2.19 (s)

Monacyclinone D (**7**, 2.2 mg) was isolated as a bright yellow powder and the molecular formula of C_27_H_30_NO_7_ ([M + H]^+^ observed: *m*/*z* 480.2022, calculated: *m*/*z* 480.2022, Δ_m_ = 0.0 ppm) was determined by HRESIMS indicating 14 double bond equivalents. Although **7** was found to have the identical molecular formula of **6**, several notable differences were found in the NMR spectra of **7** compared to **6**. The ^1^H and ^13^C NMR spectral data of **7** (Table 1) showed the presence of four aromatic protons (δ_H_ 7.53, 1H, d, *J* = 8 Hz; δ_H_ 8.01, 1H, d, *J* = 8 Hz; δ_H_ 8.05, 1H, d, *J* = 8 Hz; δ_H_ 8.09, 1H, d, *J* = 8 Hz). Most significantly, the ^1^H NMR data revealed that H-5 now appeared as a doublet (δ_H_ 7.53, *J* = 8 Hz) and was coupled with a proton at H-6 (δ_H_ 8.05, d, *J* = 8 Hz), as seen in **1** and **4**. Additionally the ^13^C spectrum showed that C-12b was considerably deshielded in **7** (Table 1), consistent with an oxygen atom bonded to this carbon. A significant difference was observed in the chemical shift of the C-1 carbonyl in **7** (δ_C_ 172.9) compared with **6** (δ_C_ 196.9), suggesting the presence of an ester or carboxylic acid functionality. In addition, **7** also contained the two methylene groups both of which appeared as an AB quartet (C-2: CH_2_ δ_H_ 3.21 d, *J*=17 Hz, 3.23 d, *J* = 17 Hz and C-4: CH_2_ δ_H_ 3.21 d, *J* = 17 Hz, 3.81 d, *J* = 17 Hz). Both also displayed HMBC correlations with the methyl substituent at C-3 as observed with **6** (Table S6; Figure 1). These results, in addition to the double bond equivalents calculated from the molecular formula, indicated that **7** contains a seven membered lactone ring formed by a Baeyer Villiger oxygenation reaction [21]. A similar angucyclinone containing a lactone moiety was identified in trace amounts from a mutant strain of *S. fradiae* [22], but our work represents the first report of such a compound obtained from a wild-type strain.

Many of the NMR spectral features of monacyclinone E (**8**, 1.8 mg) (C_27_H_32_NO_8_, [M + H]^+^ observed: *m*/*z* 498.2124, calculated: *m*/*z* 498.2128, Δ_m_ = −0.8 ppm) were similar to those of frigocyclinone (**1**) and monacyclinone D (**7**). The ^1^H and ^13^C NMR 2D spectral data of **8** (Table 1 and Table S7) indicated no changes to the aminodeoxysugar substituent, including the point of attachment at C-9 as indicated by an HMBC correlation between the aromatic carbon C-9 (δ_C_ 141.0) and the anomeric proton H-1′ (δ_H_ 5.22 m). The other spectral data (Table 1) revealed four aromatic protons H-5 (δ_H_ 8.06 d, *J* = 8 Hz), H-6 (δ_H_ 7.90 d, *J* = 8 Hz), H-10 (δ_H_ 8.13 d, *J* = 8 Hz), and H-11(δ_H_ 8.0 d, *J* = 8 Hz), on rings B and D respectively establishing the same substitution pattern as found in **1**, **4**, and **7**. The ^13^C resonance for C-1 was observed at a higher field in **8** (δ_C_ 175.7) compared with **6** (δ_C_ 196.9) (Table 1) and the chemical shift for C-12b appeared at δ_C_ 162.6, both of which suggested similarity to **7**. Based on these chemical shift changes, the loss of one double bond equivalent indicated by the molecular formula revealed that **8** was the result of an oxidative opening of the lactone ring in **7**. A similar ring opening has been observed in fridamycin D [8].

Metabolites **4**–**8** shared similar structural features with the previously reported frigocyclinone and dehydrorabelomycin. In all compounds the aminodeoxysugar was present and attached through a *C*-glycosidic bond to the C-9 of the angucyclinone chromophore. However, an additional metabolite (compound **9**) was isolated from the fermentation extract and found to have a second aminodeoxy sugar attached through an angular oxygen to the angucyclinone chromophore.

Monacyclinone F (**9**, 8.3 mg) was isolated as a purple oil and found to have the molecular formula C_35_H_47_N_2_O_10_ ([M + H]^+^ observed: *m*/*z* 655.3253, calculated: *m*/*z* 655.3231, Δ_m_ = 3.4 ppm) by HRESIMS indicating 14 double bond equivalents (Figure 2). The UV absorption spectrum of **9** showed absorption maxima at 281, 298, and 367 nm. The ^1^H and ^13^C NMR spectral data of **9** (Table 2) showed the presence of only two ortho coupled aromatic protons H-10 (δ_H_ 8.14 d, *J* = 8 Hz) and H-11 (δ_H_ 8.03 d, *J* = 8 Hz), consistent with a C/D ring system as found in **1** and **4**–**8** (Figure 2, Table 2). Once again, the chemical shift data established an aminodeoxy sugar attached in the usual fashion to C-9 of ring D as well as a phenolic group at C-8 (δ_C_ 159.2). In contrast, the protons at C-5 and C-6 now appeared as a methylene group δ_H_ 2.83 (m) and a methine δ_H_ 4.69 (m), respectively. Furthermore, the upfield chemical shift of the carbons in ring B (C-4a (δ_C_ 82.8), C-5 (δ_C_ 36.7), C-6 (δ_C_ 64.5), C-6a (δ_C_ 58.5), C-12a (δ_C_ 71.3), and C-12b (δ_C_ 80.5), established a loss of aromaticity in this ring. Thus the number of double bond equivalents must be accounted for as rings, and based on analysis of HMBC and HSQC NMR data (Table 2, Figures S18 and S19) it was found that two of the additional oxygen atoms in the molecular formula of **9** could be accounted for as epoxide rings. One of the epoxide rings included carbons C-6 (δ_C_ 64.5) and C-6a (δ_C_ 58.5), while the second contained carbons C-12a (δ_C_ 71.3) and C-12b (δ_C_ 80.5) (Table 2, Figure 1, Table S8). Further HMBC analysis revealed a strong correlation between C-4a (δ_C_ 82.8) and the anomeric proton H-1′′ (δ_H_ 5.75 s), identifying the presence of a second aminodeoxysugar (Table S8), thus accounting for the final double bond equivalent. The chemical shift of the anomeric carbon C-1′′ (δ_C_ 94.1) indicated that this sugar moiety was attached to the central core through a more common ether link and not through a *C*-glycosydic bond. There were no other significant changes to ring A as suggested by the presence of the two methylene groups at C-2 (CH_2_ δ_H_ 2.83 dd *J* = 18, 3 Hz, δ_H_ 3.34 dd *J* = 18, 3 Hz) and C-4 (CH_2_ δ_H_ 2.12 m, 2.93 dd *J* = 14, 3 Hz) that showed HMBC correlations with the methyl substituent at C-3 (Table 2; Table S8; Figure 1). The relative configuration of both aminodeoxy sugar moieties were found to be identical to that observed in frigocyclinone based on ROESY correlations between the axial hydrogen atoms as described above (Figure 1 and Figure 3).

**Table 2:** ^13^C and ^1^H NMR data for compound **9** (in pyridine-*d*_5_).

Position	^13^C	^1^H (m, *J* Hz)	COSY	HMBC	ROESY	TOCSY
1	204.4, C					
2	50.6, C	2.83 (dd, 18.0, 3.0)		4, 12a, 4a, 1, 13, 3	4	
		3.34 (dd, 18.0, 3.0)				
3	75.9, C					
4	49.8, CH_2_	2.12 (m)		13, 5, 3, 2	2	
		2.93 (dd, 14.0, 3.0)				
4a	82.8, C					
5	36.7, CH_2_	2.83 (m)	6	7a, 6, 12a, 4a, 1		6
6	64.5, CH	4.69 (m)	5			
6a	58.5, C					
7	198.0, C					
7a	118.4, C					
8	159.2, C					
9	140.7, C					
10	135.3, CH	8.14 (d, 8.0)	11	1′, 7a, 11, 11a, 8		11
11	120.5, CH	8.03 (d, 8.0)	10	7a, 9, 8, 12, 7		10
11a	133.1, C					
12	192.7, C					
12a	71.3, C					
12b	80.5, C					
13	25.7 CH_3_	1.33 (s)		4, 3, 12b, 1		4, 5
1′	65.1, CH	5.17_ax_ (m)	2′	3′, 2′, 10, 9, 8	6′, 3′_ax_	2′, 3′, 4′, 1′
2′	32.4, CH_2_	1. 26_ax_ (m)	3′, 1′	3′, 1′, 9, 4′	4′	5′, 1′, 3′
		2.22_eq_ (m)			3′_eq_	
3′	23.2, CH_2_	1.70_ax_ (m)	4′, 2′	2′, 4′	1′, 6′	2′, 6′, 4′, 5′, 1′
		1.85_eq_ (m)			2′_eq_, 5′	
4′	64.6, CH	2.36_ax_ (m)	5′, 3′	6′, 5′	2′_ax_	2′, 6′, 3′
5′	72.1, CH	4.58 (m)	6′, 4′	6′, 3′, 1′, 4′	3′_eq_	6′, 3′, 4′
6′	12.3, CH_3_	1.44_ax_ (m)	5′	4′, 5′	1′, 3′_ax_	3′, 5′
7′	43.5, CH_3_	2.25 (s)		4′		
8′	43.5, CH_3_	2.25 (s)		4′		
1′′	94.1, CH	5.75_ax_ (s)	2′′	3′′, 5′′, 4a	3′′ _ax_, 6′′	3′′, 4′′, 2′′
2′′	31.1, CH_2_	1.44_ax_ (m)	3′′, 1′′	4′′	4′′	
		2.25_eq_ (m)			3′′_eq_	
3′′	14.2, CH_2_	1.14_eq_ (m)	4′′, 2′′	5′′, 1′′, 4′′	1′′, 6′′	7′′, 4′′, 2′′, 5′′, 1′′
		1.46_ax_ (m)			2′′_eq_, 5′′	
4′′	66.1, CH	1.93_ax_ (m)	5′′, 3′′	6′′, 5′′, 3′′	2′′ _ax_	6′′, 3′′, 2′′, 5′′, 1′′
5′′	68.3, CH	3.65 (m)	6′′, 4′′	3′′, 6′′, 4′′	3′′_eq_	6′′, 3′′, 4′′, 2′′
6′′	19.1, CH_3_	0.75_ax_ (d, 6.0)	5′′	1′′, 5′′, 4′′	1′′, 3′′_ax_	3′′, 4′′, 2′′, 5′′
7′′	41.1, CH_3_	2.05 (s)		4′′		
8′′	41.1, CH_3_	2.05 (s)		4′′		

### 2.2. Cultivation of a Possible New Phylotype of Streptomyces sp.

**Figure 4:**
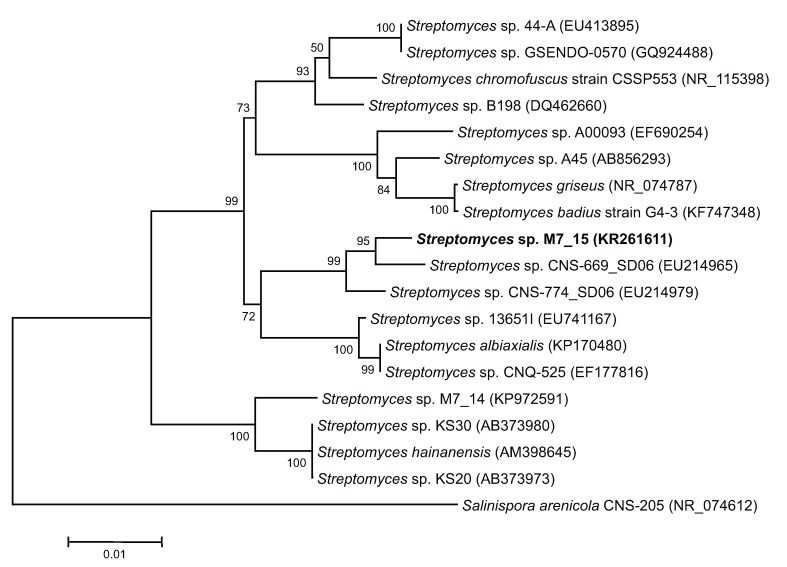
Neighbor-joining (NJ) tree of sponge and sediment associated actinomycetes based on partial sequence of 1400 base pairs of 16S rRNA genes. The tree was bootstrapped using 1000 replicates with the neighbor-joining algorithm. Scale bar = 0.01 nucleotide substitutions per site. Accession numbers of each sequence are indicated in parenthesis.

The closest relative to *Streptomyces* sp. M7_15 searched through GenBank was *Streptomyces* sp. CNS-669-SD06 with a supporting 98% sequence homology of a partial sequence of the 16S rRNA gene (Figure 4). This suggests the possibility that *Streptomyces* sp. M7_15 is a new phylotype [23] with the ability to produce new compounds, and in fact six new compounds related to frigocylinone were isolated from the EtOAc extract of the fermentation broth (Figure 2). Since frigocyclinone was previously isolated from a *Streptomyces griseus* species associated with soils of Antarctica, its partial 16S rRNA sequence was used to compare its genomic information to the 16S rRNA gene of *Streptomyces* sp. M7_15. Intriguingly, when the neighbor-joining tree was analyzed, these two strains were grouped into two separate clusters, showing a significantly distant relationship to one another (Figure 4). Therefore, it could be assumed that *Streptomyces* sp. M7_15 is a new actinomycete phylotype that has evolved similar biosynthetic genes to the Antarctic strain of *S. griseus* or that the presence of the pathways leading to angucyclinone production in one or both strains resulted from horizontal transfer and the two pathways have a common ancestor [24,25,26]. Distinguishing these two possibilities would require sequencing both pathways.

### 2.3. Biosynthetic Diversity of Angucyclinone Derivatives

Aromatic polyketides biosynthesized by a type II PKS are divided into anthracyclines, angucyclines, and tetracyclines [10,11]. Derivatives of these can undergo further oxygenation, glycosylation and dehydration and show a wide range of biological activities [12,13,21,27]. The monacyclinones isolated in this study provide examples of many such post-PKS modifications of the angucyclinone moiety, and in some cases these modifications are extremely rare. It is possible to speculate that the early biosynthetic products of this organism are frigocyclinone (**1**) or monacyclinone C (**6**) which undergo a series of further modifications. For example, either of these compounds could serve as precursors of **7** (and its hydrolysis product (**8**) following a Baeyer-Villiger oxygenation reaction. Such a reaction has been reported once in the generation of the angucyclinone derivative urdamycin L, isolated from a mutated strain of the soil bacterium *Streptomyces fradiae* [22]. Urdamycin L, which possesses a seven-membered lactone moiety in ring A [21,22], is very similar in structure to monacyclinone D (**7**) and only lacks the *C*-linked deoxyamino sugar at C-9. It is proposed that lactone production is catalyzed by the protein oxygenase UrdM [22], and might be the pathway to hydroxylation at C-12b. Certainly the discovery of the monacyclinones **7**–**8** in a wild-type strain of a *Streptomyces* sp., tends to support this proposal. It is also likely that two separate glycosyltransferase enzymes operate in the *C*- and *O*- glycosides found in monacyclinone F (**9**) as observed in other angucyclinone derivatives [11].

Another rare modification is the formation of two epoxide rings at positions 12a and 6 in monacyclinone F (**9**) and an angular oxygen at position C-4a. The placement of angular oxygens at C-12b and C-4a are widespread throughout many angucycline derivatives where the oxygens at these positions arise from acetate (C-4a) and atmospheric oxygen (C-12b) via introduction by a monooxygenase [10]. The monooxygenases responsible for these modifications are characterized as flavin-dependent monooxygenases and a recent study on these tailoring enzymes found enormous functional flexibility and the ability to catalyze alternate reactions depending on the substrate, reaction conditions, and presence of other enzymes [28,29]. Flavin-dependent monooxygenases catalyze a variety of reactions, including hydroxylation, Baeyer-Villiger oxidation, and epoxidation. The structural diversity of the monacyclinones underscores the catalytic flexibility of these tailoring enzymes resulting in the immense structural diversity of the angucycline class of natural products.

### 2.4. Biological Activity of Angucyclinone Derivatives

Frigocyclinone (**1**), exhibited weak antibiotic activity against *Bacillus subtilis* [17]. In addition, frigocyclinone showed an inhibition zone of 1 mm at a concentration of 1 mg/mL against *Mycobacterium smegmatis* (Table S9). No inhibition was observed against gram negative or fungal organisms at the concentrations tested. This compound also showed cytotoxicity against human rhabdomyosarcoma SJCRH30 cells with an EC_50_ value of 5.2 µM at 48 h post treatment (Table 3; Figures S20 and S21).

**Table 3:** EC_50_ of frigocyclinone, dimethyldehydrorabelomycin, and all new monacyclinone derivatives after 24 h and 48 h intervals against rhabdomyosarcoma cancer cells. EC_50_s are reported as >10 μM if the highest dose of the compound failed to kill 50% of the cells in that treatment.

Compound	EC_50_ 24 h (µM)	EC_50_ 48 h (µM)
Frigocyclinone (1)	>10	5.2
Dimethyldehydrorabelomycin (3)	>10	>10
4	>10	>10
5	>10	>10
6	1.1 × 10^2^	1.6 × 10^2^
7	>10	>10
8	>10	2.7 × 10^2^
9	6.1 × 10^−1^	7.3 × 10^−1^

Modifications to the angucyclinone moiety resulted in a wide variety of bioactivity profiles against the human rhabdomyosarcoma SJCRH30 cells for the monacyclinone derivatives (Figures S20 and S21; Table 3). Of all the derivatives tested, compound **9**, which has two aminodeoxysugars attached to the angucyclinone moiety, exhibited the strongest cytotoxicity against bacteria and human rhabdomyosarcoma SJCRH30 cells. This metabolite registered EC_50_ values of 610 nM and 730 nM 24 h and 48 h post treatment, respectively (Table 3). In contrast to compound **9**, compounds **4**–**8** with an aromatic ring and only one aminodeoxysugar unit showed weak to no bioactivity against gram-positive bacteria and human rhabdomyosarcoma SJCRH30 cells (Table 3; Table S9). Moreover compound **3**, that lacks both aminodeoxysugars, shows weak bioactivity in all the bioassays conducted (Table 3 and Table S9). As indicated by their structures, these bioactivity results suggest that the aminodeoxysugar subunit, the epoxide groups, and the ketone moiety could all be important for biological activity.

## 3. Experimental Section

### 3.1. General Experimental

All media components and chemicals were purchased from Fisher Scientific (Waltham, MA, USA). Optical rotation data were recorded on a Rudolph Research Analytical Autopol III automatic polarimeter (Rudolf Research, Fairfield, NJ, USA). UV spectra were recorded on a Beckman DU 640 spectrophotometer (Beckman Instruments, Fullerton, CA, USA). ^1^H NMR, COSY, HSQC, and HMBC spectra were recorded in methanol-*d*_4_ or pyridine-*d*_5_ using a 500 MHz Bruker NMR Avance spectrometer (Bruker, Billerica, MA, USA). Chromatography for LC/MS was performed using a Hewlett-Packard 1100 HPLC system equipped with a diode array detector (Hewlett-Packard, Avondale, CA, USA) using a Waters XTerra MS C18 column (2.1 × 30 mm) (Waters, Milford, MA, USA) and a gradient mobile phase of MeCN/H_2_O each containing 0.1% acetic acid. A Waters Micromass ZQ detector (Waters, Milford, MA, USA) equipped with an electrospray ionization source was used to acquire mass spectrometric data (capillary voltage 3.5 kV; cone voltages 30 V/50 V; source temperature 100 °C; desolvation temperature 400 °C; cone gas flow 60 L/h; desolvation gas flow 450 L/h). All samples were run in both positive and negative ion mode with a mass range of 100–1400 Da and a scan time of 0.5 s The LC/MS data were analyzed using MassLynx 4.1 (Waters, Milford, MA, USA). High-resolution mass measurements were outsourced to the University of Illinois SCS Mass Spectrometry Lab (Urbana, IL, USA). HPLC was conducted on a Waters 1525 HPLC (Waters, Milford, MA, USA) coupled with a Waters 2487 dual λ absorbance detector (Waters, Milford, MA, USA). The UV wavelengths used for separations were 214 and 250 nm. The flow rate of the mobile phase was 4 mL/min for 10 mm columns and 0.8 mL/min for 4.6 mm columns.

### 3.2. Isolation of Streptomyces *sp.* M7_15

Strain *Streptomyces* sp. M7_15 was isolated from the Caribbean marine sponge *Scopalina ruetzleri.* The sponge *S. ruetzleri* was collected by SCUBA at a depth of 20 m on Las Carmelitas dive site located in Mona Island, Puerto Rico (18°6′25.72″ N, 67°56′11.35″ W). A volume of 1 mL of sponge tissue was scraped from the coral bottom with a sterile razor blade and transferred to a 50-mL sterile tube with sterilized tweezers. Sterile mortars and pestles were used to homogenize the sponge before dilution. The sponge homogenate was then treated with heat (55 °C for 60 min) and agitation (vortexed for 1 min) in order to select for actinomycetes. Media M6 (1 L 75% ocean seawater, 500 mg mannitol, 100 mg peptone and 18 g agar [30]) was used to isolate *Streptomyces* sp. M7_15. M6 media was supplemented with 50 μg/mL of the fungicide cyclohexamide and 20 μg/mL of the antibiotic novobiocin to facilitate the isolation of actinobacteria and deter the growth of sponge associated fungi and gram-negative bacteria on the plates [30,31]. Aliquots of 100 μL were transferred from the *S. ruetzleri* homogenate dilution to the M6 media. After 8 weeks of incubation of the sponge homogenate from *Scopalina ruetzleri*, the actinomycete strain *Streptomyces* sp. M7_15 appeared as a brown circular colony that produced white spores and a dark purple diffusible pigment. The strain is kept in culture on agar slants as well as cryopreserved.

### 3.3. DNA Extraction, Sequencing and Phylogenetic Analysis

DNA was extracted from a 20 mg pellet of cells from a pure isolate using the Gentra Puregene kit (Qiagen, Valencia, CA, USA). The 16S rRNA gene was amplified using polymerase chain reaction (PCR) with universal eubacterial primers 27F and 1492R [32,33]. The PCR mix consisted of 39.3 μL of nuclease free water, 0.2 μL of Platinum Taq DNA Polymerase High Fidelity (Invitrogen, Carlsbad, CA, USA), 1 μL (40 ng/μL) of each primer, and 1 μL (100 ng/μL) of DNA template. The PCR program was set to an initial denaturation at 95 °C for 2.5 min, followed by 35 cycles of 95 °C for 30 s, 45 °C for 30 s, 72 °C for 1 min, and a final extension during the last cycle of 72 °C for 10 min. Results of PCR reactions were observed on 1% agarose gels. Sequencing reactions were conducted with 685R primer and Big Dye terminator (Applied Biosystems, Foster City, CA, USA). The reaction products were analyzed with a 3130XL Genetic Analyzer (Applied Biosystems, Foster City, CA, USA). Sequences were assembled and edited using Sequencher 5.2.4 (Gene Codes, Ann Arbor, MI, USA). Double coverage reads allowed for accurate editing of sequences. The complete consensus sequence was exported into a single Fasta file from Sequencher and uploaded with MEGA 5 (Gene Codes, Ann Arbor, MI, USA) [34]. Sequences were then aligned using the ClustalW function with default parameters (ClustalW, University College Dublin, Dublin, Ireland). The closest relative to our sequence was searched using the BLAST function from GenBank. These sequences were used as reference sequences for alignment and tree generating purposes. MEGA 5 was used to construct a phylogenetic tree of the 16S rRNA gene using a neighbor-joining (NJ) analysis. The NJ analysis was performed using a maximum composite likelihood model of nucleotide substitution and data was re-sampled using 1000 bootstrap replicates.

### 3.4. Chemical Analysis of Actinomycetes

*Streptomyces* sp. M7_15 was fermented in large scale in P3C media (10 g starch, 4 g yeast, 2 g peptone, 1 g CaCO_3_, 40 mg Fe_2_SO_4_, 100 mg KBr, in 1 L of 75% seawater) over a period of 3 months. Batches (1.6 L total volume) of Streptomyces sp. M7_15 were cultured at 28 °C on a rotary shaker (IKA, Wilmington, NC, USA) in 500 mL flasks containing 100 mL of media each during a period of 9 days on a rotary shaker (200 rpm). Cells and fermented broth were harvested by vacuum filtration using glass fiber filters (GF/C) (Whatman, Maidstone, UK). Media was extracted by liquid-liquid partitioning using ethyl acetate (EtOAc) and butanol (BuOH) (2:1). The cells retained on the glass fiber filter were extracted with 80% aq.MeOH. Extracts were monitored by LC/MS and tested for antimicrobial activities against gram positive (*Bacillus subtilis* and *Mycobacterium smegmatis*), gram negative (*Escherechia coli*) and fungal (*Aspergillus niger* and *Candida kefyr*) organisms.

### 3.5. Purification of Monacyclinones from Streptomyces *sp.* M7_15

LC/MS analysis and antibiotic assays of the crude EtOAc extract (986 mg) showed potent bioactivity and displayed characteristic UV spectral data (190 nm and 234 nm). Subsequently, this extract was suspended in 1.5 mL of 20% aqueous MeOH and was fractionated using a reversed-phase C_18_ Sep-pak (10 g) column (Waters Associates, Milford, MA, USA) with a step gradient of MeOH/H_2_O elution. Seven fractions were collected starting at 20% MeOH with increments of 20% MeOH followed by dichloromethane (CH_2_Cl_2_) and 0.1% TFA in MeOH washes. LC-MS and antibiotic activity of the fractions indicated that the antimicrobial compounds were distributed in the 80%–100% MeOH fractions with the highest concentration in 80% MeOH (306 mg). Subsequent fractionation was performed using a reversed-phase C_18_ Baker Bond (6 g) column (Baker-bond, Philipsburg, NJ, USA) and a stepwise elution gradient of MeOH/H_2_O starting with 60% MeOH. Fractions were collected according to the color of the bands as they eluted from the column. A total of 14 fractions were collected and three fractions (70 mgs each) following 80% MeOH elution showed the most potent activity. These fractions were further purified by HPLC using a semiprep C_18_ column (Sun Fire, Petaluma, CA, USA; 10 × 250 mm, 5 µm) with a gradient mobile phase (20%–70% MeOH over 28 min). Active fractions obtained from this step were further fractionated using an analytical C_18_ column (Sun Fire, Petaluma, CA, USA; 4.6 × 150 mm, 3.5 µm) and a gradient mobile phase 30%–45% acetonitrile (ACN). This final fractionation process yielded 1–10 mg of each of 8 active compounds that were eventually identified as frigocyclinone (**1**), dimethyldehydrorabelomycin (**3**) and monacyclinones A–F (**4**–**9**).

Frigocyclinone (**1**): Yellow powder; ^1^H NMR (500 MHz) and ^13^C NMR (125 MHz,) see Table S1; HRESIMS (+) *m*/*z* 464.2061 (calcd for C_21_H_17_O_5_ 464.2073 ∆ = −2.6 ppm).

Dimethyldehydrorabelomycin (**3**): Red powder, ${\left[\text{α}\right]}_{\text{D}}^{\text{25}}$ 63° (*c* 0.9, DMSO); UV (Pyridine) λ_max_ (log ε) 318 (3.6), 290 (3.44), 415 (3.21); ^1^H NMR (500 MHz) and ^13^C NMR (125 MHz) see Table S2; HRESIMS (+) *m*/*z* 349.1071 (calcd for C_21_H_17_O_5_ 349.1076 ∆ = −1.4 ppm).

Monacyclinone A (**4**): yellow powder, ${\left[\text{α}\right]}_{\text{D}}^{\text{25}}$ 50° (*c* 1.8, DMSO); UV (Pyridine) λ_max_ (log ε) 315 (3.44), 280 (3.12), 422 (3.0); ^1^H NMR (500 MHz) and ^13^C NMR (125 MHz) see Table S3; HRESIMS (+) *m*/*z* 446.1974 (calc for C_27_H_28_NO_5_ 446.1967 ∆ = 1.6 ppm).

Monacyclinone B (**5**): orange powder, ${\left[\text{α}\right]}_{\text{D}}^{\text{25}}$ 43° (*c* 1.0, DMSO); UV (Pyridine) λ_max_ (log ε) 328 (3.19), 299 (4.9), 445 (3.13); ^1^H NMR (500 MHz) and ^13^C NMR (125 MHz) see Table S4; HRESIMS (+) *m*/*z* 462.1917 (calc for C_27_H_28_NO_6_ 462.1917 ∆ = 0.0 ppm).

Monacyclinone C (**6**): yellow powder, ${\left[\text{α}\right]}_{\text{D}}^{\text{25}}$ 15° (*c* 1.5, DMSO); UV (Pyridine) λ_max_ (log ε) 280 (3.21), 429 (2.98), 301 (2.91); ^1^H NMR (500 MHz) and ^13^C NMR (125 MHz) see Table S5; HRESIMS (+) *m*/*z* 480.2027 (calc for C_27_H_30_NO_7_ 480.2022 ∆ = 1.0 ppm).

Monacyclinone D (**7**): yellow powder, ${\left[\text{α}\right]}_{\text{D}}^{\text{25}}$ 9° (*c* 1.5, DMSO); UV (Pyridine) λ_max_ (log ε) 440 (3.44), 285 (3.36), 404 (3.28); ^1^H NMR (500 MHz) and ^13^C NMR (125 MHz) see Table S6; HRESIMS (+) *m*/*z* 480.2022 (calc for C_27_H_30_NO_7_ 480.2022 ∆ = 0.0 ppm).

Monacyclinone E (**8**): yellow powder, ${\left[\text{α}\right]}_{\text{D}}^{\text{25}}$ 12° (*c* 1.5, DMSO); UV (Pyridine) λ_max_ (log ε) 440 (3.44), 285 (3.36), 404 (3.28); ^1^H NMR (500 MHz) and ^13^C NMR (125 MHz) see Table S7; HRESIMS (+) *m*/*z* 498.2124 (calc for C_27_H_32_NO_8_ 498.2128 ∆ = −0.8 ppm).

Monacyclinone F (**9**): purple oil, ${\left[\text{α}\right]}_{\text{D}}^{\text{25}}$ 80° (*c* 3.5, DMSO); UV (Pyridine) λ_max_ (log ε) 367 (3.18), 281 (314), 298 (3.00); ^1^H NMR (500 MHz) and ^13^C NMR (125 MHz) see Table S8; HRESIMS (+) *m*/*z* 655.3253 (calc for C_35_H_47_N_2_O_10_ 655.3231 ∆ = 3.44 ppm).

### 3.6. Image Based Cytotoxicity Assay Using SJCRH30 (Rhabdomyosarcoma) Cells

Cytotoxicity was assessed using a live cell imaged based assay that utilizes the fluorescent nuclear stain Hoechst 33342 (H-dye) (Sigma, St Louis, MO, USA) and the fluorescent microtubule stain Tubulin Tracker 488 (Life Technologies, Gaithersburg, MD, USA) [35]. For this assay, the most sensitive cell line for marine toxins was found to be the SJCRH30 cells. In addition to being very sensitive to marine toxins, these cells are large and spread out which allows for counting efficiency for live cell imaging and allowed for easier measurement and visualization of morphological changes associated with toxin. Comparison of the half maximal effective concentration (EC_50_) values for the traditional XTT cytotoxicity assay and the *in vitro* microscopy assay using the human cell line model (SJCHR30) showed no significant difference between the assays [35], therefore the *in vitro* cytotoxicity assay was used to determine the EC_50_ values for the marine derived products in this manuscript.

Cytotoxicity was measured using fluorescence staining of cell nucleii. Initially, SJCRH30 cells were seeded at a density of 5000 cells/well in BD biocoat poly-d-lysine-coated 96-well plates (Becton Dickinson, Franklin Lakes, NJ, USA) and incubated at 37 °C overnight. Cells were then treated with 10× solutions of compounds added directly to the growth medium (RPMI 1640 supplemented with 10% FBS) and incubated at 37 °C for the designated time period. Cell nucleii were stained with a 0.1 µg/mL final concentration of Hoechst 33342 (Invitrogen, Grand Island, NY, USA) at the same time as the compound treatment. 10 µL of 10× Hoechst dye in Phosphate Buffered Saline was added directly to the growth medium in the wells, and the plate was incubated at 37 °C for the remaining time. Cells had to be stained in two separate steps because Hoechst 33,342 is not compatible with the Hanks Buffered Saline Solution (HBSS) that is required for the tubulin staining protocol.

To stain microtubules with Tubulin Tracker 488 (TT488), a 500 µM intermediate stock was first prepared by combining 1 mM TT488 (Invitrogen, Camarillo, CA, USA) with an equal volume of 20% Pluronic F-127 in DMSO (Invitrogen, Camarillo, CA, USA). Then a 200 nM TT488 staining solution was prepared from the intermediate stock in HBSS. The growth medium containing the Hoechst dye was removed from the wells, and the cells were rinsed once with warm HBSS (100 µL per well). The HBSS rinse was replaced with warmed 200 nM TT488 staining solution (100 µL per well). The plate was then returned to the 37 °C incubator for 20 min before imaging on an Image Xpress Micro system (Molecular Devices, Sunnyvale, CA, USA), equipped with an environmental control chamber warmed to 37 °C. The intensity of the fluorescence of the Hoechst dye stain was measured on the DAPI channel and that of the TT488 was measured on the FITC channel of the Image Xpress Micro (Molecular Devices, Sunnyvale, CA, USA) using the MetaXpress Image Acquisition and Analysis v2.0.1.44 software (Molecular Devices, Sunnyvale, CA, USA). The percentage of DAPI positively-stained cells as compared to control treatments (percentage of maximum cell survival) averaged within 4 snapshots of each well were calculated with SAS v9.1.3 software (SAS Institute Inc., Cary, NC, USA) and analyzed with non-linear regression curve-fit analysis by GraphPad Prism v4.03 (Graphpad, San Diego, CA, USA) to yield EC_50_ values. In addition, pictures of cells from the cytotoxicity experiments stained with Hoechst dye and Tubulin Tracker were visualized using the MetaXpress Image Acquisition and Analysis v2.0.1.44 software (Molecular Devices, Sunnyvale, CA, USA).

EC_50_ values were determined using an ANOVA SAS v9.1.3 software (SAS Institute Inc., Cary, NC, USA). In all experiments, results are presented as the mean ± SD and were considered statistically significant if a *p*-value was less than 0.05.

## 4. Conclusions

This study describes six new angucyclinone derivatives which, in addition to frigocyclinone, add to the list of angucyclinones with a *C*-linked aminodeoxysugar. Several novel features include the second appearance of a lactone ring as part of the angucyclinone moiety (compound **7**) and the addition of a second aminodeoxysugar with two epoxide rings (compound **9**). These compounds are an example of the remarkable biosynthetic ability of marine sponge-associated *Streptomyces* sp. to produce bioactive secondary metabolites. Despite the weak biological activities of monacyclinones A–E (**4**–**8**), monacyclinone F (**9**) with an additional aminodeoxysugar moiety showed potent bioactivity against rhabdomyosarcoma SJCRH30 cells. These bioactivity profiles should encourage further bioassays of angucyclinone derivatives against other cancer cell lines.

## Supplementary Files

Supplementary File 1

## Abbreviations

NMR: Nuclear Magnetic Resonance
COSY: Correlation Spectroscopy
HMBC: Heteronuclear Multiple-Bond Connectivity
ROESY: Rotating frame Nuclear Overhauser Spectroscopy
HSQC: Heteronuclear Single Quantum Coherence
TOCSY: Total Correlation Spectroscopy
HPLC: High Performance Liquid Chromatography
LC/MS: Liquid Chromatography/Mass Spectrometry
