# Supplementary material for: Monacyclinones, New Angucyclinone Metabolites Isolated from Streptomyces sp. M7_15 Associated with the Puerto Rican Sponge Scopalina ruetzleri
Source: Mar Drugs. 2015 Jul 29;13(8):4682–700. doi: 10.3390/md13084682 (PMC4556999; doi:10.3390/md13084682)
Supplement: Supplementary File 1 [file marinedrugs-13-04682-s001.docx]

Supplementary Materials

| Table S1. ^13^C and ^1^H NMR data for compound **1** (in methanol-*d*_4_). | S2 |
| --- | --- |
| Table S2. ^13^C and ^1^H NMR data for compound **3** (in pyridine-*d*_5_). | S2 |
| Table S3. ^13^C and ^1^H NMR data for compound **4** (in pyridine-*d*_5_). | S3 |
| Table S4. ^13^C and ^1^H NMR data for compound **5** (in pyridine-*d*_5_). | S4 |
| Table S5. ^13^C and ^1^H NMR data for compound **6** (in pyridine-*d*_5_). | S5 |
| Table S6. ^13^C and ^1^H NMR data for compound **7** (in pyridine-*d*_5_). | S5 |
| Table S7. ^13^C and ^1^H NMR data for compound **8** (in pyridine-*d*_5_). | S6 |
| Table S8. ^13^C and ^1^H NMR data for compound **9** (in pyridine-*d*_5_). | S7 |
| Figure S1. ^1^H NMR spectrum of **4** (in pyridine-*d*_5_). | S8 |
| Figure S2. ^1^H–^1^H COSY spectra of **4** (in pyridine-*d*_5_). | S9 |
| Figure S3. ^1^H–^13^C HMBC spectra of **4** (in pyridine-*d*_5_). | S9 |
| Figure S4. ^1^H NMR spectrum of **5** (in pyridine-*d*_5_). | S10 |
| Figure S5. ^1^H–^1^H COSY spectra of **5** (in pyridine-*d*_5_). | S10 |
| Figure S6. ^1^H–^13^C HMBC spectra of **5** (in pyridine-*d*_5_). | S11 |
| Figure S7. ^1^H NMR spectrum of **6** (in pyridine-*d*_5_). | S11 |
| Figure S8. ^1^H–^1^H COSY spectra of **6** (in pyridine-*d*_5_). | S12 |
| Figure S9. ^1^H–^13^C HMBC spectra of **6** (in pyridine-*d*_5_). | S12 |
| Figure S10. ^1^H NMR spectrum of **7** (in pyridine-*d*_5_). | S13 |
| Figure S11. ^1^H–^1^H COSY spectra of **7** (in pyridine-*d*_5_). | S13 |
| Figure S12. ^1^H–^13^C HMBC spectra of **7** (in pyridine-*d*_5_). | S14 |
| Figure S13. ^1^H NMR spectrum of **8** (in pyridine-*d*_5_). | S14 |
| Figure S14. ^1^H–^1^H COSY spectra of **8** (in pyridine-*d*_5_). | S15 |
| Figure S15. ^1^H–^13^C HMBC spectra of **8** (in pyridine-*d*_5_). | S15 |
| Figure S16. ^1^H NMR spectrum of **9** (in pyridine-*d*_5_). | S16 |
| Figure S17. ^1^H–^1^H COSY spectra of **9** (in pyridine-*d*_5_). | S16 |
| Figure S18. ^1^H–^13^C HSQC spectra of **9** (in pyridine-*d*_5_). | S17 |
| Figure S19. ^1^H–^13^C HMBC spectra of **9** (in pyridine-*d*_5_). | S17 |
| Table S9. Antibiotic activity of **1**, **3**, **4**–**9.** | S18 |
| Figure S20. Cytotoxicity of **1**, **3**, **4**–**9** after 24 h. | S18 |
| Figure S21. Cytotoxicity of **1**, **3**, **4**–**9** after 48 h. | S18 |

**Table S1.** ^13^C and ^1^H NMR data for compound **1** (in *d*_4_**-**methanol).

| **Position** | **^13^C** | **^1^H (m, *J* Hz)** | **COSY** | **HMBC** |
| --- | --- | --- | --- | --- |
| 1 | 199.1, C |  |  |  |
| 2 | 54.6, CH_2_ | 3.07 (m) |  | 3, 1 |
|  |  | 2.87 (m) |  |  |
| 3 | 72.9, C |  |  |  |
| 4 | 44.9, CH_2_ | 3.21 (d, 17.0) |  | 2, 3, 4a, 12b |
|  |  | 3.12 (d, 17.0) |  |  |
| 4a | 150.4, C |  |  |  |
| 5 | 135.3, CH | 7.60 (d, 8.0) | 6 | 12b, 6a |
| 6 | 130.2, CH | 8.20 (d, 8.0) | 5 | 12a, 4a |
| 6a | 134.7, C |  |  |  |
| 7 | 184.2, C |  |  |  |
| 7a | 115.8, C |  |  |  |
| 8 | 159.4, C |  |  |  |
| 9 | 138.4, C |  |  |  |
| 10 | 134.6, CH | 7.79 d (8.0) | 11 | 8, 11a, 1′ |
| 11 | 120.0, CH | 7.51 d (8.0) | 10 | 7a, 9, 12 |
| 11a | 134.6, C |  |  |  |
| 12 | 184.2, C |  |  |  |
| 12a | 136.8, C |  |  |  |
| 12b | 136.6, C |  |  |  |
| 13 | 30.1, CH_3_ | 1.42 (s) |  | 4, 2, 3 |
| 1′ | 65.6, CH | 5.07 d (11.0) | 2′ | 10, 9, 8 |
| 2′ | 32.6, CH_3_ | 2.25 (d,14.0) | 3′ |  |
|  |  | 1.42 (m) |  |  |
| 3′ | 22.4, CH_2_ | 2.15 (d, 10.0) | 4′ |  |
|  |  | 1.89 (m) |  |  |
| 4′ | 65.8, CH | 2.93 (m) | 5′ |  |
| 5′ | 71.1, CH | 4.58 (m) | 4′ |  |
| 6′ | 12.3, CH_3_ | 1.42 (m) |  |  |
| 7′ | 43.1, CH_3_ | 2.61 (s) |  | 4′ |
| 8′ | 43.1, CH_3_ | 2.61 (s) |  | 4′ |

**Table S2.** ^13^C and ^1^H NMR data for compound **3** (in pyridine-*d*_5_).

| **Position** | **δ ^13^C** | **δ ^1^H (m, *J* Hz)** | **COSY** | **HMBC** | **TOCSY** |
| --- | --- | --- | --- | --- | --- |
| 1 | 158.3, C |  |  |  |  |
| 2 | 109.2, CH | 6.76 (s) |  | 12b, 1, 4 |  |
| 3 | 142.1, C |  |  |  |  |
| 4 | 119.1, CH | 7.10 (s) |  | 2, 12b, 4a |  |
| 4a | 140.8, C |  |  |  |  |
| 5 | 117.2, CH | 7.57 (s) |  | 12b, 6a, 6 |  |
| 6 | 157.8, C |  |  |  |  |
| 6a | 120.4, C |  |  |  |  |

**Table S2.** *Cont.*

| 7 | 190.1, C |  |  |  |  |
| --- | --- | --- | --- | --- | --- |
| 7a | 120.5, C |  |  |  |  |
| 8 | 160.9, C |  |  |  |  |
| 9 | 117.9, CH | 7.28 (d, 8.0) | 10 | 7a, 11, 8 | 10, 11 |
| 10 | 136.8, CH | 7.69 (dd, 8.0, 8.0) | 9, 11 | 8, 11a | 9, 11 |
| 11 | 118.9, CH | 7.81 d (8.0) | 10 | 7a, 9, 12 | 9, 10 |
| 11a | 139.8, C |  |  |  |  |
| 12 | 187.8, C |  |  |  |  |
| 12a | 137.8, C |  |  |  |  |
| 12b | 116.2, C |  |  |  |  |
| 13 | 22.3, CH_3_ | 2.36 (s) |  | 2, 4, 3 |  |
| 14 | 57.0, CH_3_ | 3.91 (s) |  | 8 |  |
| 15 | 56.5, CH_3_ | 3.96 (s) |  | 1 |  |

**Table S3.** ^13^C and ^1^H NMR data for compound **4** (in pyridine-*d*_5_).

| **Position** | **δ ^13^C** | **δ ^1^H (m, *J* Hz)** | **COSY** | **HMBC** | **ROESY** | **TOCSY** |
| --- | --- | --- | --- | --- | --- | --- |
| 1 | 156.7, C |  |  |  |  |  |
| 2 | 119.9, CH | 7.33 (s) |  | 1, 4, 13 | 13 | 13, 4 |
| 3 | 142.6, C |  |  |  |  |  |
| 4 | 121.6, CH | 7.28 (s) |  | 5, 12b, 4a | 5, 13 | 13, 2 |
| 4a | 120.3, C |  |  |  |  |  |
| 5 | 137.5, CH | 8.19 (d, 8.0) | 6 | 4, 6a | 6, 4 | 6 |
| 6 | 122.5, CH | 8.40 (d, 8.0) | 5 | 12a, 7 | 5 | 5 |
| 6a | 135.6, C |  |  |  |  |  |
| 7 | 188.1, C |  |  |  |  |  |
| 7a | 115.0, C |  |  |  |  |  |
| 8 | 158.7, C |  |  |  |  |  |
| 9 | 140.0, C |  |  |  |  |  |
| 10 | 134.1, CH | 8.03 (d, 8.0) | 11 | 1′, 11a, 8 | 11 | 11 |
| 11 | 121.4, CH | 7.91 (d, 8.0) | 10 | 7a, 9, 12 | 10 | 10 |
| 11a | 134.6, C |  |  |  |  |  |
| 12 | 189.5, C |  |  |  |  |  |
| 12a | 134.3, C |  |  |  |  |  |
| 12b | 121.1, C |  |  |  |  |  |
| 13 | 21.5, CH_3_ | 2.39 (s) |  | 4, 3, 2 |  | 2, 4 |
| 1′ | 65.2, CH | 5.26_ax_ (d, 11.0) | 2′ | 9 | 6′, 3′_ax_ | 4′, 2′, 3′, 5′ |
| 2′ | 32.5, CH_2_ | 1.44_ax_ (m) | 1′, 3′ |  | 4′ | 3′, 4′, 1′ |
|  |  | 2.39_eq_ (m) |  |  | 3′_eq_ |  |
| 3′ | 22.4, CH_2_ | 1.98_ax_ (m) | 4′, 2′ |  | 1′, 6′ | 4′, 2′, 1′, 5′ |
|  |  | 1.98_eq_ (m) |  |  | 2′_eq_, 5′ |  |
| 4′ | 64.7, CH | 2.82_ax_ (br s) | 5′, 3′ |  | 2′_ax_ | 5′, 2′, 1′, 3′ |
| 5′ | 71.2, CH | 4.72 (m) | 6′, 4′ | 6′, 3′, 1′ | 3′_eq_ | 6′,4′, 3′, 2′ |

**Table S3.** *Cont.*

| 6′ | 12.4, CH_3_ | 1.52_ax_ (d, 7.0) | 5′ | 4′, 5′ | 1′, 3′_ax_ | 5′ |
| --- | --- | --- | --- | --- | --- | --- |
| 7′ | 42.8, CH_3_ | 2.52 (s) |  | 4′ |  |  |
| 8′ | 42.8, CH_3_ | 2.52 (s) |  | 4′ |  |  |

**Table S4.** ^13^C and ^1^H NMR data for compound **5** (in pyridine-*d*_5_).

| **Position** | **δ ^13^C** | **δ ^1^H (m, *J* Hz)** | **COSY** | **HMBC** | **ROESY** | **TOCSY** |
| --- | --- | --- | --- | --- | --- | --- |
| 1 | 164.8, C |  |  |  |  |  |
| 2 | 116.0, CH | 8.22 (s) | 4, 13 | 1, 4, 4a, 13 | 13 | 4, 13 |
| 3 | 141.5, C |  |  |  |  |  |
| 4 | 121.0, CH | 7.43 (s) | 2, 13 | 2, 4a, 5, 13 | 13 | 2, 13 |
| 4a | 124.2, C |  |  |  |  |  |
| 5 | 157.4, C |  |  |  |  |  |
| 6 | 105.6, CH | 8.01 (s) |  | 7, 4a, 12a |  |  |
| 6a | 123.5, C |  |  |  |  |  |
| 7 | 189.9, C |  |  |  |  |  |
| 7a | 115.2, C |  |  |  |  |  |
| 8 | 158.9, C |  |  |  |  |  |
| 9 | 139.9, C |  |  |  |  |  |
| 10 | 134.0, CH | 8.05 (d, 8.0) | 11 | 1′, 11a, 8 |  | 11 |
| 11 | 121.3, CH | 7.97 (d, 8.0) | 10 | 7a, 9, 12 |  | 10 |
| 11a | 135.1, C |  |  |  |  |  |
| 12 | 187.5, C |  |  |  |  |  |
| 12a | 132.2, C |  |  |  |  |  |
| 12b | 123.7, C |  |  |  |  |  |
| 13 | 21.7, CH_3_ | 2.46 (s) | 2, 4 | 2, 4, 3 |  | 2, 4 |
| 1′ | 65.2, CH | 5.23_ax_ (m) | 2′ | 9 | 6′, 3′_ax_ | 4′, 2′, 3′, 5′ |
| 2′ | 32.5, CH_2_ | 1.44_ax_ (m) | 3′, 1′ | 5′, 4′ | 4′ | 3′, 4′, 1′ |
|  |  | 2.30_eq_ (m) |  |  | 3′_eq_ |  |
| 3′ | 22.8, CH_2_ | 1.79_ax_ (m) | 4′, 2′ |  | 1′, 6′ | 4′, 2′, 1′, 5′ |
|  |  | 1.93_eq_ (m) |  |  | 2′_eq_, 5′ |  |
| 4′ | 64.5, CH | 2.53_ax_ (s) | 5′, 3′ |  | 2′_ax_ | 5′, 2′, 1′, 3′ |
| 5′ | 71.6, CH | 4.64 (m) | 6′, 4′ | 6′, 3′, 4′ | 3′_eq_ | 6′,4′, 3′, 2′ |
| 6′ | 12.2, CH_3_ | 1.44_ax_ (m) | 5′ | 5′ | 1′, 3′_ax_ | 5′ |
| 7′ | 43.2, CH_3_ | 2.31 (s) |  | 4′ |  |  |
| 8′ | 43.2, CH_3_ | 2.31 (s) |  | 4′ |  |  |

**Table S5.** ^13^C and ^1^H NMR data for compound **6** (in pyridine-*d*_5_).

| **Position** | **δ ^13^C** | **δ ^1^H (m, *J* Hz)** | **COSY** | **HMBC** | **ROESY** | **TOCSY** |
| --- | --- | --- | --- | --- | --- | --- |
| 1 | 196.9, C |  |  |  |  |  |
| 2 | 54.5, CH_2_ | 3.22 (m) 3.22 (m) |  | 1, 13, 4, 3, 12b, 4a | 13, 4 | 13, 4 |
| 3 | 71.9, CH_2_ |  |  |  |  |  |
| 4 | 45.0, CH_2_ | 3.25 (m)  3.25 (m) |  | 2, 12b, 3, 13 | 13, 2 | 13, 2 |
| 4a | 153.6, C |  |  |  |  |  |
| 5 | 122.4, CH | 7.16 (s) |  | 4, 6a, 12a, 6, 7 |  |  |
| 6 | 164.5, C |  |  |  |  |  |
| 6a | 117.5, C |  |  |  |  |  |
| 7 | 193.1, C |  |  |  |  |  |
| 7a | 115.4, C |  |  |  |  |  |
| 8 | 158.8, C |  |  |  |  |  |
| 9 | 139.0, C |  |  |  |  |  |
| 10 | 134.5, CH | 8.03 (d, 8.0) | 11 | 1′, 11a, 8 | 11 | 11 |
| 11 | 119.5, CH | 7.82 (d, 8.0) | 10 | 7a, 9, 12 |  | 10 |
| 11a | 135.3, C |  |  |  |  |  |
| 12 | 184.1, C |  |  |  |  |  |
| 12a | 130.6, C |  |  |  |  |  |
| 12b | 122.6, C |  |  |  |  |  |
| 13 | 30.1, CH_3_ | 1.56 (s) |  | 4, 2, 3 | 2, 4 | 2, 4 |
| 1′ | 65.1, CH | 5.22_ax_ (dd, 11.0, 1.0) | 2′ | 10, 9, 8 | 6′, 3′_ax_ | 4′, 2′, 3′, 5′ |
| 2′ | 32.1, CH_2_ | 1.36_ax_ (m) | 3′, 1′ | 1′ | 4′ | 3′, 4′, 1′ |
|  |  | 2.34_eq_ (m) |  |  | 3′_eq_ |  |
| 3′ | 22.7, CH_2_ | 1.81_ax_ (m) | 4′, 2′ | 4′, 2′ | 1′, 6′ | 4′, 2′, 1′, 5′ |
|  |  | 1.94_eq_ (m) |  |  | 2′_eq_, 5′ |  |
| 4′ | 64.7, CH | 2.53_ax_ (m) | 5′, 3′ |  | 2′_ax_ | 5′, 2′, 1′, 3′ |
| 5′ | 71.9, CH | 4.61 (m) | 6′, 4′ | 6′, 3′, 4′ | 3′_eq_ | 6′,4′, 3′, 2′ |
| 6′ | 12.3, CH_3_ | 1.44_ax_ (d, 7.0) | 5′ | 4′, 5′ | 1′, 3′_ax_ | 5′ |
| 7′ | 43.0, CH_3_ | 2.34 (s) |  | 4′ |  |  |
| 8′ | 43.0, CH_3_ | 2.34 (s) |  | 4′ |  |  |

**Table S6.** ^13^C and ^1^H NMR data for compound **7** (in pyridine-*d*_5_).

| **Position** | **δ ^13^C** | **δ ^1^H (m, *J* Hz)** | **COSY** | **HMBC** | **ROESY** | **TOCSY** |
| --- | --- | --- | --- | --- | --- | --- |
| 1 | 172.9, C |  |  |  |  |  |
| 2 | 45.3, CH_2_ | 3.23 (d, 17.0)  3.21 (d, 17.0) |  | 1, 3, 4, 13 | 13 | 13, 4 |
| 3 | 90.7, C |  |  |  |  |  |
| 4 | 40.3, CH_2_ | 3.21 (d, 17.0) |  | 2, 3, 4a, 12b, 13 | 13 | 13, 2 |
|  |  | 3.81 (d, 17.0) |  | 2, 3, 4a, 12b, 13 |  |  |
| 4a | 139.4, C |  |  |  |  |  |
| 5 | 130.5, CH | 7.53 (d, 8.0) | 6 | 4, 6a, 12b |  | 6 |
| 6 | 120.8, CH | 8.01 (d, 8.0) | 5 | 7, 12a, 4a |  | 5 |

**Table S6.** *Cont.*

| 6a | 133.4, C |  |  |  |  |  |
| --- | --- | --- | --- | --- | --- | --- |
| 7 | 189.6, C |  |  |  |  |  |
| 7a | 115.9, C |  |  |  |  |  |
| 8 | 159.2, C |  |  |  |  |  |
| 9 | 138.2, C |  |  |  |  |  |
| 10 | 133.7, CH | 8.09 (d, 8.0) | 11 | 1′, 11a, 8 | 11 | 11 |
| 11 | 119.0, CH | 8.05 (d, 8.0) | 10 | 7a, 9, 12 | 10 | 10 |
| 11a | 133.4, C |  |  |  |  |  |
| 12 | 181.0, C |  |  |  |  |  |
| 12a | 116.7, C |  |  |  |  |  |
| 12b | 160.0, C |  |  |  |  |  |
| 13 | 27.3, CH_3_ | 1.78 (s) |  | 2, 3, 4 |  | 2, 4 |
| 1′ | 64.7, CH | 5.26_ax_ (m) | 2′ | 9 | 6′, 3′_ax_ | 4′, 2′, 3′, 5′ |
| 2′ | 31.9, CH_2_ | 1.39_ax_ (m) | 3′, 1′ | 4′ | 4′ | 3′, 4′, 1′ |
|  |  | 2.33_eq_ (m) |  |  | 3′_eq_ |  |
| 3′ | 22.5, CH_2_ | 1.83_ax_ (m) | 4′, 2′ |  | 1′, 6′ | 4′, 2′, 1′, 5′ |
|  |  | 1.94_eq_ (m) |  |  | 2′_eq_, 5′ |  |
| 4′ | 64.2, CH | 2.54_ax_ (m) | 5′, 3′ |  | 2′_ax_ | 5′, 2′, 1′, 3′ |
| 5′ | 71.8, CH | 4.63 (m) | 6′, 4′ | 1′, 3′, 4′, 5′ | 3′_eq_ | 6′,4′, 3′, 2′ |
| 6′ | 12.0, CH_3_ | 1.46_ax_ (d, 7.0) | 5′ | 4′, 5′ | 1′, 3′_ax_ | 5′ |
| 7′ | 43.0, CH_3_ | 2.33 (s) |  | 4′ |  |  |
| 8′ | 43.0, CH_3_ | 2.33 (s) |  | 4′ |  |  |

**Table S7.** ^13^C and ^1^H NMR data for compound **8** (in pyridine-*d*_5_).

| **Position** | **δ ^13^C** | **δ ^1^H (m, *J* Hz)** | **COSY** | **HMBC** | **ROESY** | **TOCSY** |
| --- | --- | --- | --- | --- | --- | --- |
| 1 | 175.7, C |  |  |  |  |  |
| 2 | 46.9, CH_2_ | 3.02 (m)  3.02 (m) |  | 13, 4, 3, 1 | 13 | 13, 4 |
| 3 | 72.4, C |  |  |  |  |  |
| 4 | 41.0, CH_2_ | 2.52 (m)  3.47 (m) |  | 13, 2, 3, 5, 12b, 4a |  | 13, 2 |
| 4a | 136.7, C |  |  |  |  |  |
| 5 | 140.7, CH | 8.06 (d, 8.0) | 6 | 4, 6a, 12a, 12b | 6, 4 | 6a, 4 |
| 6 | 118.9, CH | 7.90 (d, 8.0) | 5 | 5, 7, 4a, 12a | 5 | 5 |
| 6a | 132.4, C |  |  |  |  |  |
| 7 | 188.9, C |  |  |  |  |  |
| 7a | 116.3, C |  |  |  |  |  |
| 8 | 160.0, C |  |  |  |  |  |
| 9 | 141.0, C |  |  |  |  |  |
| 10 | 134.0, CH | 8.13 (d, 8.0) | 11 | 1′, 11a, 8 | 11 | 11 |
| 11 | 119.6, CH | 8.00 (d, 8.0) | 10 | 7a, 9, 12 |  | 10 |
| 11a | 132.6, C |  |  |  |  |  |

**Table S7.** *Cont.*

| 12 | 189.1, C |  |  |  |  |  |
| --- | --- | --- | --- | --- | --- | --- |
| 12a | 116.3, C |  |  |  |  |  |
| 12b | 162.6, C |  |  |  |  |  |
| 13 | 27.8, CH_3_ | 1.69 (s) |  | 4, 2, 3 |  | 2, 4 |
| 1′ | 65.2, CH | 5.25_ax_ (m) | 2′ | 2′, 10, 9, 8 | 6′, 3′_ax_ | 4′, 2′, 3′, 5′ |
| 2′ | 32.2, CH_2_ | 1.42_ax_ (m) | 3′, 1′ | 1′ | 4′ | 3′, 4′, 1′ |
|  |  | 2.33_eq_ (m) |  |  | 3′_eq_ |  |
| 3′ | 23.1, CH_2_ | 1.69_ax_ (m) | 2′, 4′ |  | 1′, 6′ | 4′, 2′, 1′, 5′ |
|  |  | 1.89_eq_ (m) |  |  | 2′_eq_, 5′ |  |
| 4′ | 64.7, CH | 2.33_ax_ (m) | 3′, 5′ | 4′, 5′, 6′ | 2′_ax_ | 5′, 2′, 1′, 3′ |
| 5′ | 72.0, CH | 4.61 (m) | 6′ | 6′, 3′, 4′ | 3′_eq_ | 6′,4′, 3′, 2′ |
| 6′ | 12.3, CH_3_ | 1.42_ax_ (m) | 5′ | 4′, 5′ | 1′, 3′_ax_ | 5′ |
| 7′ | 43.3, CH_3_ | 2.19 (s) |  | 4′ |  |  |
| 8′ | 43.3, CH3 | 2.19 (s) |  | 4′ |  |  |

**Table S8.** ^13^C and ^1^H NMR data for compound **9** (in pyridine-*d*_5_).

| **Position** | **δ ^13^C** | **δ ^1^H (m, *J* Hz)** | **COSY** | **HMBC** | **ROESY** | **TOCSY** |
| --- | --- | --- | --- | --- | --- | --- |
| 1 | 204.4, C |  |  |  |  |  |
| 2 | 50.6, CH_2_ | 2.83 (dd, 18.0, 3.0)  3.34 (dd, 18.0, 3.0) |  | 4, 12a, 4a, 1, 13, 3 |  | 4, 13 |
| 3 | 75.9, C |  |  |  |  |  |
| 4 | 49.8, CH_2_ | 2.12 (m)  2.93 (dd, 14.0, 3.0) |  | 13, 3, 2 | 2 | 2, 13 |
| 4a | 82.8, C |  |  |  |  |  |
| 5 | 36.7, CH_2_ | 2.83 (m) | 6 | 6a, 6, 12a, 4a, 1, 4 | 6 | 6 |
| 6 | 64.5, CH | 4.69 (m) | 5 | 5, 6a, 12a, 7 |  | 5 |
| 6a | 58.5, C |  |  |  |  |  |
| 7 | 198.0, C |  |  |  |  |  |
| 7a | 118.4, C |  |  |  |  |  |
| 8 | 159.2, C |  |  |  |  |  |
| 9 | 140.7, C |  |  |  |  |  |
| 10 | 135.3, CH | 8.14 (d, 8.0) | 11 | 1′, 11a, 8 | 11 | 11 |
| 11 | 120.5, CH | 8.03 (d, 8.0) | 10 | 7a, 9, 8, 12 |  | 10 |
| 11a | 133.1, C |  |  |  |  |  |
| 12 | 192.7, C |  |  |  |  |  |
| 12a | 71.3, C |  |  |  |  |  |
| 12b | 80.5, C |  |  |  |  |  |
| 13 | 25.7, CH_3_ | 1.33 (s) |  | 4, 3, 2, 1 |  | 4, 2 |
| 1′ | 65.1, CH | 5.17_ax_ (m) | 2′ | 3′, 2′, 10, 9, 8 | 6′, 3′_ax_ | 2′, 3′, 4′ |
| 2′ | 32.4, CH_2_ | 1.26_ax_ (m) | 3′, 1′ | 3′, 1′, 9, 4′ | 4′ | 1′, 3′, 5′ |
|  |  | 2.22_eq_ (m) |  |  | 3′_eq_ |  |

**Table S8.** *Cont.*

| 3′ | 23.2, CH_2_ | 1.70_ax_ (m) | 4′, 2′ | 2′, 4′ | 1′, 6′ | 2′, 6′, 4′, 1′ |
| --- | --- | --- | --- | --- | --- | --- |
|  |  | 1.85_eq_ (m) |  |  | 2′_eq_, 5′ |  |
| 4′ | 64.6, CH | 2.36_ax_ (m) | 5′, 3′ | 6′, 5′ | 2′_ax_ | 2′, 6′, 3′, 5′ |
| 5′ | 72.1, CH | 4.58 (m) | 6′, 4′ | 6′, 3′, 1′, 4′ | 3′_eq_ | 6′, 3′ |
| 6′ | 12.3, CH_3_ | 1.44_ax_ (m) | 5′ | 4′, 5′ | 1′, 3′_ax_ | 3′ |
| 7′ | 43.5, CH_3_ | 2.25 (s) |  | 4′ |  |  |
| 8′ | 43.5, CH_3_ | 2.25 (s) |  | 4′ |  |  |
| 1′′ | 94.1, CH | 5.75_ax_ (s) | 2′′ | 3′′, 5′′, 4a | 3′′_ax_, 6′′ | 3′′, 4′′, 2′′ |
| 2′′ | 31.1, CH_2_ | 1.44_ax_ (m) | 3′′, 1′′ | 4′′ | 4′′ | 3′′ |
|  |  | 2.25_eq_ (m) |  |  | 3′′_eq_ |  |
| 3′′ | 14.2, CH_2_ | 1.14_eq_ (m) | 4′′, 2′′ | 5′′, 1′′, 4′′ | 1′′, 6′′ | 6′′, 4′′, 2′′, 1′′ |
|  |  | 1.46_ax_ (m) |  |  | 2′′_eq_, 5′′ |  |
| 4′′ | 66.1, CH | 1.93_ax_ (m) | 5′′, 3′′ | 6′′, 5′′, 3′′ | 2′′ _x_ | 6′′, 3′′, 2′′, 5′′, 1′′ |
| 5′′ | 68.3, CH | 3.65 (m) | 6′′, 4′′ | 3′′, 6′′, 4′′ | 3′′_eq_ | 6′′, 3′, 2′′ |
| 6′′ | 19.1, CH_3_ | 0.75_ax_ (d, 6.0) | 5′′ | 1′′, 5′′, 4′′ | 1′′, 3′′_ax_ | 1′′, 3′′, 4′′, 2′′ |
| 7′′ | 41.1, CH_3_ | 2.05 (s) |  | 4′′ |  |  |
| 8′′ | 41.1, CH_3_ | 2.05 (s) |  | 4′′ |  |  |

**Figure S1.** ^1^H NMR spectrum of **4** (in pyridine-*d*_5_).

**Figure S2.** ^1^H–^1^H COSY spectra of **4** (in pyridine-*d*_5_).

**Figure S3.** ^1^H–^13^C HMBC spectra of **4** (in pyridine-*d*_5_).

**Figure S4.** ^1^H NMR spectrum of **5** (in pyridine-*d*_5_).

**Figure S5.** ^1^H–^1^H COSY spectra of **5** (in pyridine-*d*_5_).

**Figure S6.** ^1^H–^13^C HMBC spectra of **5** (in pyridine-*d*_5_).

**Figure S7.** ^1^H NMR spectrum of **6** (in pyridine-*d*_5_).

**Figure S8.** ^1^H–^1^H COSY spectra of **6** (in pyridine-*d*_5_).

**Figure S9.** ^1^H–^13^C HMBC spectra of **6** (in pyridine-*d*_5_).

**Figure S10.** ^1^H NMR spectrum of **7** (in pyridine-*d*_5_).

**Figure S11.** ^1^H–^1^H COSY spectra of **7** (in pyridine-*d*_5_).

**Figure S12.** ^1^H–^13^C HMBC spectra of **7** (in pyridine-*d*_5_).

**Figure S13.** ^1^H NMR spectrum of **8** (in pyridine-*d*_5_).

**Figure S14.** ^1^H–^1^H COSY spectra of **8** (in pyridine-*d*_5_).

**Figure S15.** ^1^H–^13^C HMBC spectra of **8** (in pyridine-*d*_5_).


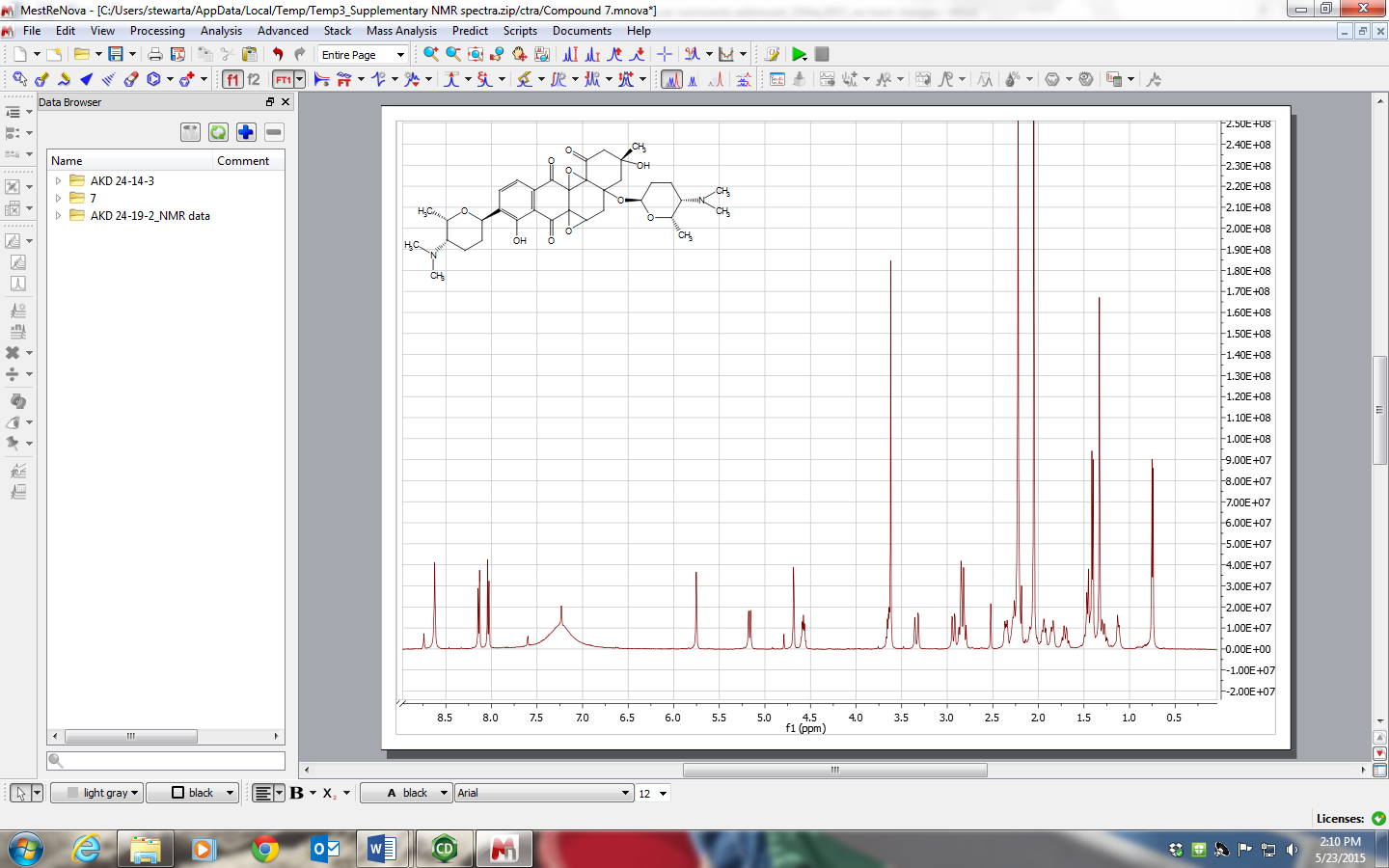


**Figure S16.** ^1^H NMR spectrum of **9** (in pyridine-*d*_5_).


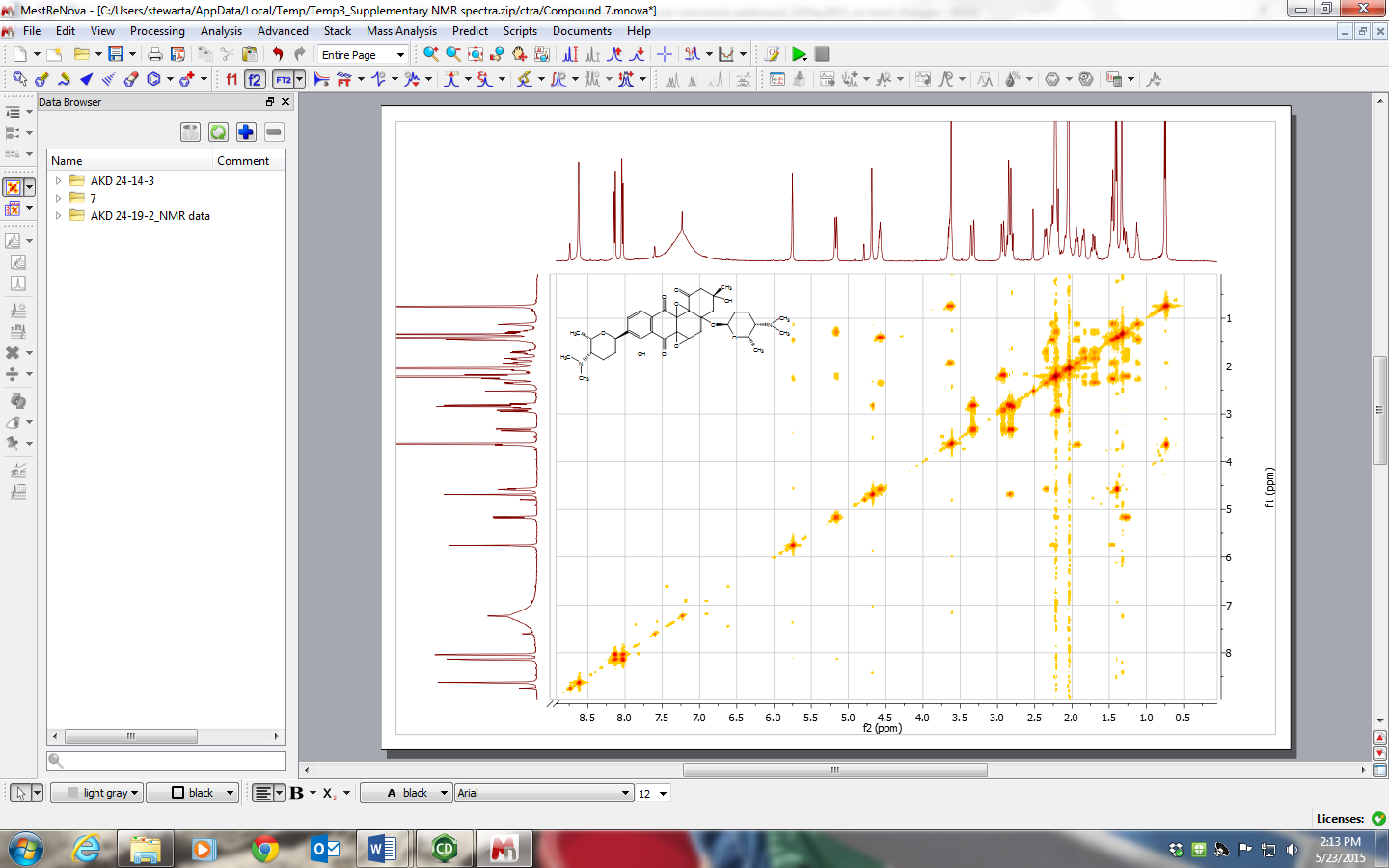


**Figure S17.** ^1^H–^1^H COSY spectra of **9** (in pyridine-*d*_5_).


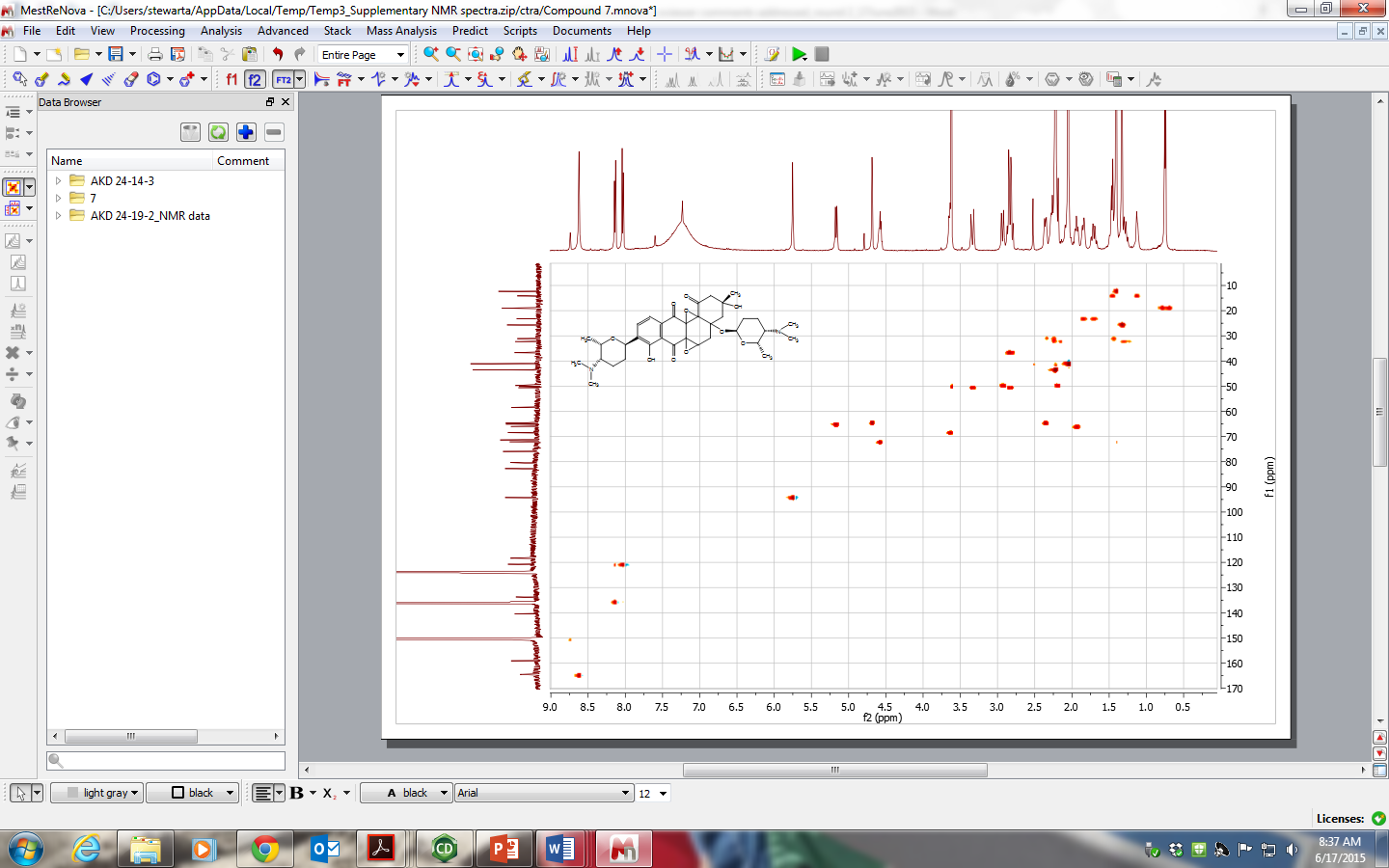


**Figure S18.** ^1^H–^13^C HSQC spectra of **9** (in pyridine-*d*_5_).


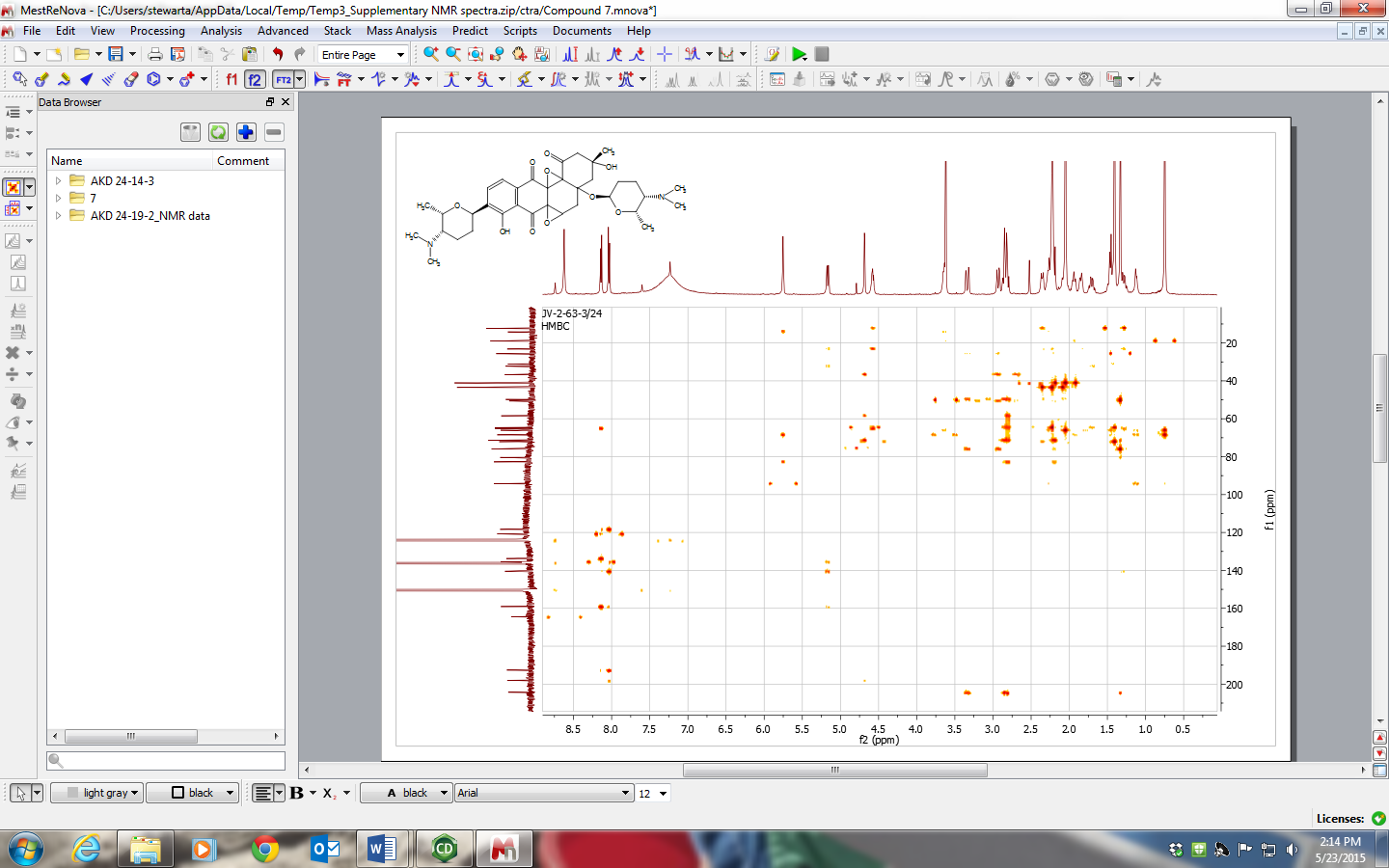


**Figure S19.** ^1^H–^13^C HMBC spectra of **9** (in pyridine-*d*_5_).

**Table S9.** Antibiotic activity of **1**, **3**, and **4**–**9**.

| **Compound Tested (1 mg/mL)** | ***Bacillus Subtilis* Inhibition Diameter (mm)** | ***Mycobacterium Smegmatis* Inhibition Diameter (mm)** |
| --- | --- | --- |
| Frigocyclinone (**1**) | 1 | 1 |
| Dimethyldehydrorabelomycin (**3**) | 2 | 2 |
| Monacyclinone A (**4**) | 1 | 1 |
| Monacyclinone B (**5**) | 2 | 2 |
| Monacyclinone C (**6**) | 3 | 3 |
| Monacyclinone D (**7**) | 2 | 2 |
| Monacyclinone E (**8**) | 2 | 2 |
| Monacyclinone F (**9**) | 4 | 4 |


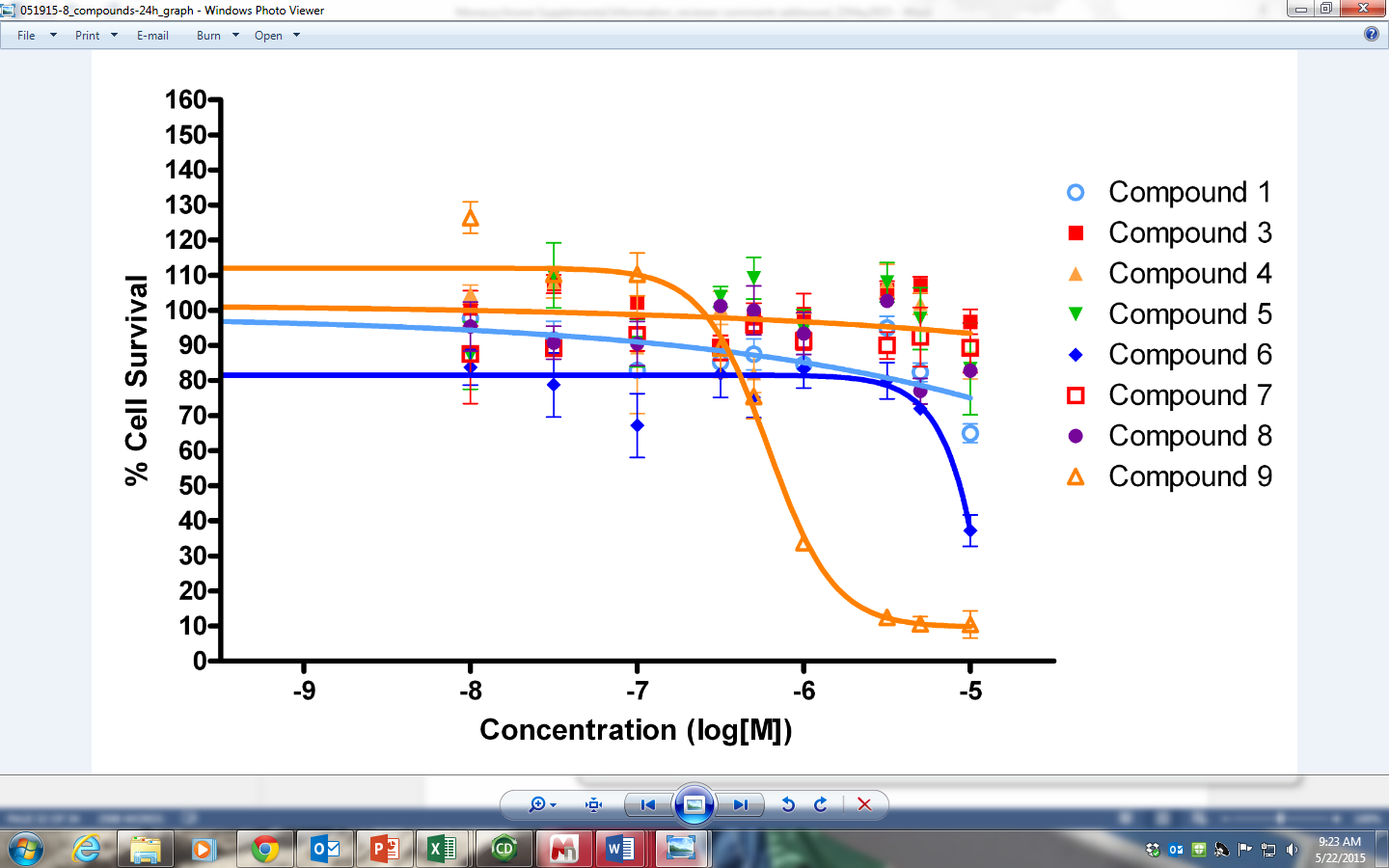


**Figure S20.** Dose response curves for 7 new monacyclinone derivatives in the SJCRH30 rhabdomyosarcoma cell cytotoxicity assay at 24 h post treatment. Each point represents the mean ± standard deviation for *n* = 3 replicates.


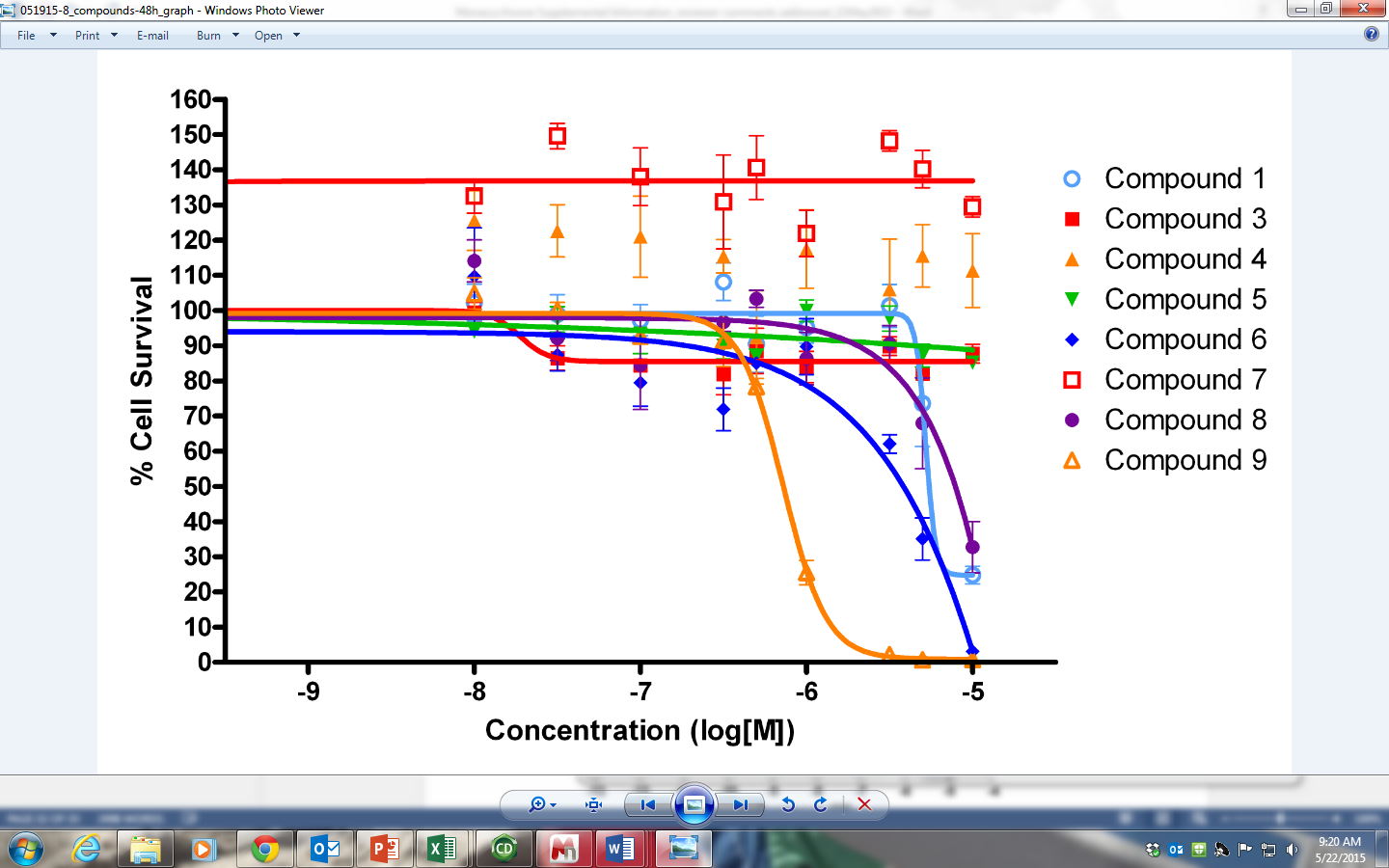


**Figure S21.** Dose response curves for 7 new monacyclinone derivatives in the SJCRH30 rhabdomyosarcoma cell cytotoxicity assay at 48 h post treatment. Each point represents the mean ± standard deviation for *n* = 3 replicates.

© 2015 by the authors; licensee MDPI, Basel, Switzerland. This article is an open access article distributed under the terms and conditions of the Creative Commons Attribution license (http://creativecommons.org/licenses/by/4.0/).
